# Which Adverse and Positive Childhood Experiences are most Strongly Associated with Young Peoples’ Arrests and Convictions?

**DOI:** 10.1007/s42844-026-00205-7

**Published:** 2026-03-30

**Authors:** Jaime La Charite, Adam Schickedanz, Christopher Biely, Rebecca Dudovitz, Alma D. Guerrero, Kathryn M. Leifheit, Annette Marinello, Benjamin P. L. Meza, Shirley Russ, Narayan Sastry, George M. Slavich, Elizabeth S. Barnert

**Affiliations:** 1 Division of General Internal Medicine and Health Services Research, Department of Medicine; David Geffen School of Medicine at UCLA, 1100 Glendon Ave. Suite 900, Los Angeles, CA 90024 USA; 2 Department of Health Policy and Management, UCLA Fielding School of Public Health, Fielding School of Public Health, University of California, 650 Charles E Young Drive, Los Angeles, CA 90095 USA; 3 Department of Pediatrics, David Geffen School of Medicine at UCLA, 10833 LeConte Ave., 12-358 CHS, Los Angeles, CA 90095 USA; 4 David Geffen School of Medicine at UCLA, 10833 LeConte Ave., 12-358 CHS, Los Angeles, CA 90095 USA; 5 Center for Healthier Children, Families, and Communities, University of California, 10960 Wilshire Blvd, Ste 960, Los Angeles, CA 90024 USA; 6 Institute for Social Research, University of Michigan, Ann Arbor. Michigan; 426 Thompson St, Ann Arbor, MI 48104 USA; 7 Department of Psychiatry and Biobehavioral Sciences, University of California, 760 Westwood Plaza, Los Angeles, CA 90095 USA

**Keywords:** Adverse childhood experience, Positive childhood experiences, Criminal justice, Juvenile legal system, Sex differences

## Abstract

Adverse childhood experiences (ACEs) are associated with young people’s criminal legal system involvement, but the extent to which distinct types of ACEs and positive childhood experiences (PCEs) differentially relate to arrests and convictions for youth is unknown. We identified childhood exposures that remain independently associated with arrests and convictions by age 26 among a broad set of ACEs and PCEs. We conducted a cross-sectional analysis of data from two components of the nationally representative Panel Study of Income Dynamics. We examined the occurrence and number of arrests and convictions by age 26 and constructed ACE- and PCE-type indicator variables and overall scores. Weighted, covariate-adjusted logistic and negative binomial regression models estimated associations between ACE types and arrests and conviction outcomes, controlling for PCE score. Similarly, we estimated PCE-type outcome associations controlling for ACE score. Finally, we examined sex differences and stratified results by race. Of 7,200 participants, 14.9% were arrested by age 26. Controlling for PCE score, physical abuse and parental divorce/separation were associated with both arrests and convictions. Arrests were also associated with sexual abuse and parental substance use. Convictions were also associated with emotional abuse and parental mental health problems. Experiencing more PCEs was protective against arrest. We found sex differences for children experiencing sexual abuse. Certain ACEs appear more closely linked to young people’s risk of arrests and convictions, while combined PCE types may be protective. Interventions and policies that target these specific ACEs and boost PCEs may help disrupt pathways into the criminal legal system.

## Key Points

Childhood exposure to physical abuse and parental divorce/separation were associated with both arrests and convictions before age 26.

Greater exposure to cumulative positive childhood experiences across multiple domains was associated with reduced likelihood of arrests before age 26.

There are gender and racial differences in the association between different types of adverse childhood experiences and legal system contact.

## Introduction

Adverse childhood experiences (ACEs) – typically categorized as abuse (physical, emotional, or sexual), neglect, or household dysfunction or challenges – are associated with poor health over the life course (Felitti et al., 1998; Hughes et al., 2017; Petruccelli et al., 2019). ACEs are common among youth involved in the carceral system and are strongly related to young people’s risk of contact with the juvenile and adult criminal legal system (herein, “legal system”) and recidivism (Baglivio et al. 2014a, b; Graf et al. 2021; Yohros 2023). In contrast, emerging evidence suggests that composite measures of positive childhood experiences (PCEs) – defined as supportive parental, school, and neighborhood relationships and environments - may reduce a young person’s risk of legal system involvement even in the presence of adversity (Baglivio & Wolff, 2021). Although composite measures of ACEs and PCEs are associated with a young person’s risk of and protection from criminal legal system involvement, respectively, the relative association between specific ACEs and PCEs and young people’s arrests and convictions is unclear (Baglivio & Wolff, 2021; Graf et al., 2021; Testa et al., 2022).

While composite ACE and PCE scores – which is how ACEs and PCEs are typically reported - provide a multidimensional risk assessment for poor legal system outcomes at the population level, they lack precision and can obscure the individual contributions of specific ACEs and PCEs, thereby limiting insights into the specific pathways through which victimization escalates to legal system contact (Graf et al., 2021). A more granular analysis that isolates which particular ACEs and PCEs remain independently associated with legal outcomes, while controlling for concurrent adversities and protective factors, could elucidate the root factors driving youth involvement in the legal system. Such insights could guide the development of tailored interventions, particularly in resource-limited settings where focused strategies may be more efficient. For instance, preventing or responding to intimate partner violence versus child physical abuse can require distinct interventions (Centers for Disease Control and Prevention, 2019; Niolon et al., 2017; Poole et al., 2014). Similarly, interventions could also be targeted based on whether limited resources are directed toward promoting PCEs in homes, neighborhoods, or schools.

Previous studies examining the relationships between childhood experiences and legal system outcomes has been limited by key methodological constraints, including reliance on composite ACE and PCE scores, examination of only a narrow subset of exposures, evaluation of risk and protective factors outside the traditional ACE and PCE frameworks, and use of samples that are not nationally representative of U.S. youth (Barnert et al., 2021; Craig et al., 2017; Sivertsson et al., 2023; Testa et al., 2022). The available literature suggests that individual protective and risk factors are important to consider beyond cumulative exposure (Aazami et al., 2023; Barnert et al., 2021; Farrington et al., 2016; Sivertsson et al., 2023; Sparks et al., 2021). However, to our knowledge, no prior studies have evaluated the risks of legal system outcomes for young people using a comprehensive set of ACE and PCE measures while simultaneously controlling for many early childhood social exposures to identify which ones remain significant within a nationally representative U.S. sample. A risk and protective factor analysis that compares a broad set of ACEs and PCEs simultaneously and identifies which childhood experiences remain independently associated with arrest and conviction outcomes for young people may identify opportunities to interrupt the primary pathways contributing to early legal system involvement, with potential benefits for health and well-being across the life course.

Disaggregating ACEs also allows exploration of how specific experiences may differentially affect subgroups by race and gender, which is essential for addressing disparities in legal system involvement. Although the original set of ACEs was identified in a predominantly white, middle-to-upper-class, privately-insured sample, composite and individual types of ACEs disproportionally impact racial/ethnic minoritized groups (Mersky et al., 2021; Slopen et al., 2016). According to the CDC’s ACE Pyramid and the Culturally-Informed ACE Framework, ACE exposure and its sequelae are one mechanism through which to understand how structural determinants of health (e.g., historical trauma) and racism-informed social conditions and stressors contribute to differential poor health outcomes (Bernard et al., 2020; Center for Disease Control and Prevention, 2021). Despite the strong impact of systemic racism on the criminal justice system, it is not well established whether the link between ACE types and legal system involvement differs by racial group; for instance, due to variable access to supports to respond to ACE exposure (Baglivio et al. 2014a, b).

Gender-specific impacts may also be present. Gendered pathways research theorizes that there are distinct pathways for females and males as they engage with the legal system (Nuytiens & Christiaens, 2016). According to strain theory, this may be due to gendered socialization in sources of stress and coping styles (Broidy & Agnew, 1997). Compared to boys, girls are also more likely to be arrested for status offenses – behaviors that are illegal because of the youth’s age - like running away (Chesney-Lind & Merlo, 2015; Kruzan & Lesley, 2025; Spivak et al., 2014). Status offenses, like many juvenile criminal legal system charges, are associated with trauma exposure (Jones & Pierce, 2021; Kruzan & Lesley, 2025; McGill & Stefurak, 2021). The ACE literature suggests that girls may be more sensitive to certain ACEs compared to boys, such as parental marital discord, which shows differential impacts on mental health and delinquency behaviors (Brock & Kochanska, 2016; Essex et al., 2003; Zahn et al., 2010). Another salient ACE is childhood sexual abuse, as girls – including those involved in the legal system – are known to have higher rates of sexual abuse, compared to boys (Baglivio et al. 2014a, b). In a recent report of adults currently incarcerated for crimes committed as children, the rate of childhood sexual abuse was as high as 80% for females compared to 45% for males (Lesley & Pierre, 2025), which is about 5–10% higher than prior studies of separate female and male samples of incarcerated adults reporting a history of childhood sexual abuse (Johnson et al., 2006; White & Frisch-Scott, 2023). It remains unclear, though, if sexual abuse has a differential influence on arrest rates by gender while controlling for other types of ACEs, PCEs, and relevant characteristics.

To address these gaps, we conducted a cross-sectional analysis using a nationally representative sample. Our primary aim was to investigate associations between individual ACE and PCE types and arrest and conviction outcomes by young adulthood. As a secondary exploratory aim, we examined whether these associations varied by race and by gender for sexual abuse. Because the ACE and PCE frameworks are being used to guide policy and clinical decision-making, it is important to develop a granular understanding of which types of ACEs and PCEs to prioritize and which subgroups to target for further investigation and intervention planning to disrupt legal system involvement into young adulthood.

## Method

### Study Design, Setting, and Participants

We conducted a cross-sectional risk and protective factor analysis using nationally representative data from a U.S. panel survey that in 2014 asked adult respondents (ages 18–97) to retrospectively report on their childhood (< 17 years) experiences and arrests and convictions before young adulthood (defined here as < 26 years) in alignment with developmental science for transitional aged youth (Wilens & Rosenbaum, 2013). The represented childhood period (birth cohorts) includes the years 1917–1996. The STROBE guidelines for reporting observational studies were used to prepare this manuscript (STROBE, 2023).

## Data Sources

### Panel Study of Income Dynamics (PSID)

We obtained data from PSID, a national household panel survey that has been collecting annual or biennial survey data on individuals and families since 1968. The original 1968 sample of 4,802 families was formed from two independent samples: an oversample comprising 1,872 low-income families from the Survey of Economic Opportunity and a separate nationally representative sample of 2,930 families. Together, the two samples formed a national probability sample of U.S. families in 1968. Anyone who was subsequently born to or adopted by a sample person is incorporated into the sample. Sample members are also followed when they create separate family units. To maintain national representativeness, PSID added 500 immigrant families to the sample in 1997 (Beaule et al., 2015).

We drew the analytic sample from the 2014 PSID Childhood Retrospective Circumstance Study (PSID-CRCS) (McGonagle & Freedman, 2015). We obtained individual-level demographic information from the 2013 Core PSID 2013 interview using the sample members’ unique identification number (Beaule et al., 2015).

### PSID-CRCS

We drew arrest, conviction, ACE, and PCE information from the 2014 PSID-CRCS, which asked adults to retrospectively report on their childhood experiences, arrests, and convictions. Individuals were eligible for PSID-CRCS if they were the financially responsible person, or their spouse/partner, in a family unit that completed the 2013 Core PSID survey in English. The number of eligible cases was 12,985. Eligible individuals were invited to participate with an invitation letter to complete the PSID-CRCS online. Non-respondents were sent biweekly reminders through email and postal mail, received telephone calls after six weeks, and were subsequently sent a paper questionnaire. Response rates were high in both the 2013 Core PSID and PSID-CRCS; however, PSID-CRCS had lower response rates for respondents who were younger and from PSID’s immigrant and low-income oversamples compared to others in the 2013 Core PSID sample (McGonagle & Freedman, 2015). Sample weights were based on the 2013 Core PSID cross-sectional individual weights and accounted for differential probabilities of selection and non-response to CRCS, including the observed differences in response patterns (Beaule et al., 2015). Details are provided in technical reports.

PSID-CRCS was completed by 8,072 adults (weighted response rate of 67%, similar to other national panel web-based surveys) (McGonagle & Freedman, 2015). PSID participants are provided an incentive for each wave of data collection in which they complete an interview. Although the survey was fielded in 2014, it is the most timely known data source that allowed us to answer our research question in a nationally representative sample. It also includes adults of all ages - offering a historical perspective that is not available from more recent data sources. Despite the wide age range of participants (18–97 years), we chose to retain the intact PSID-CRCS sample to preserve power and maintain a national representative sample of adults of all ages.

### 2013 Core PSID Survey

We obtained demographic covariate information from the 2013 Core PSID Survey. This survey collected nationally representative data on 14,562 adults through telephone interviews with 9,569 households (response rate 91%) (Beaule et al., 2015).

### Analytic Sample

**Fig. 1:**
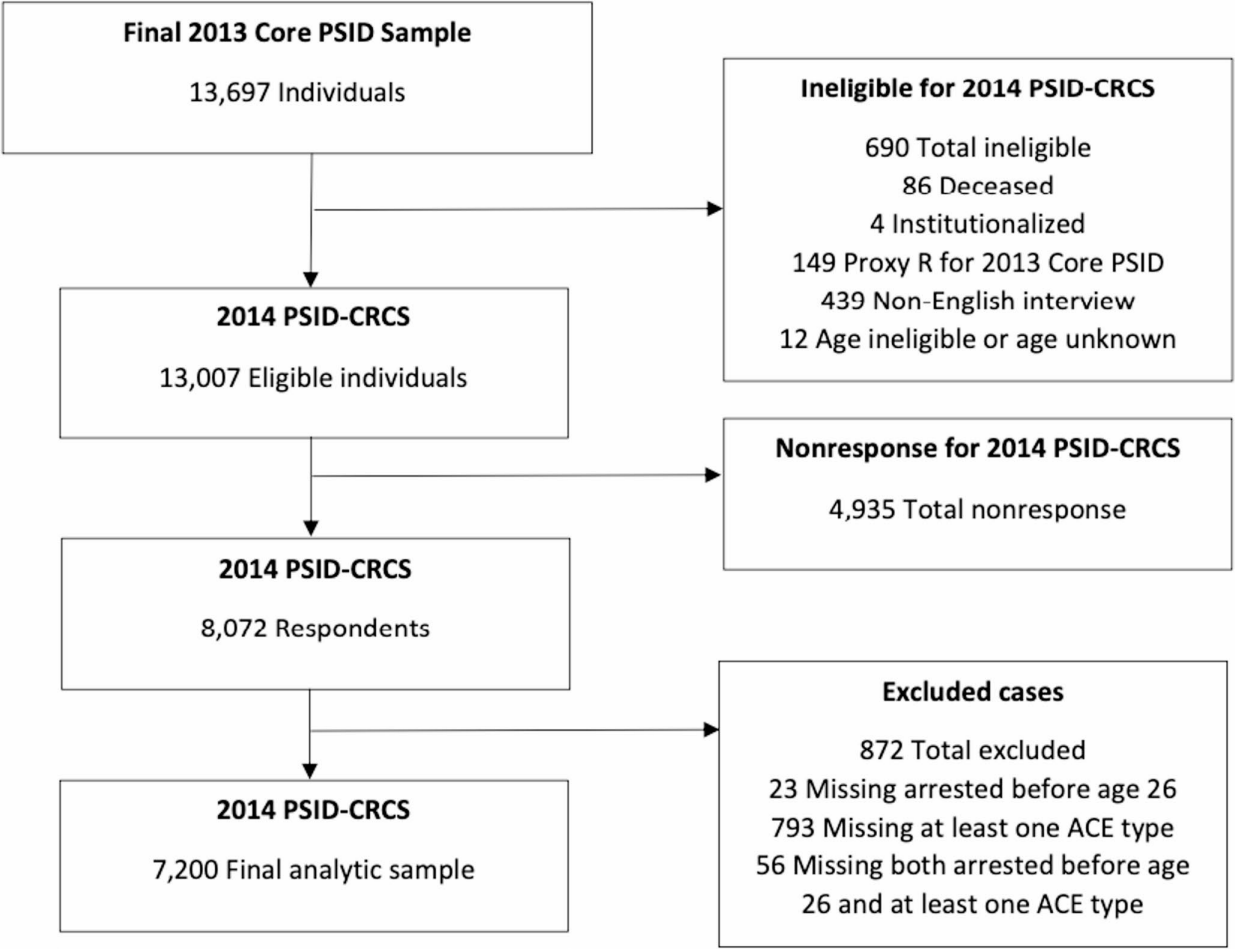
Study sample flow diagram

We defined our analytic sample as adults who completed the PSID-CRCS and had complete data on the primary outcome (arrests) and ACE types (see Fig. 1).

## Measures

### Dependent Variable-Arrest and Conviction Outcomes

PSID-CRCS respondents self-reported their arrests and convictions. The primary outcome was being arrested at least once before age 26. Respondents were asked, “Before you were age 26, were you ever arrested or taken into custody by the police?” They were then asked, “How many times were you arrested before you were 26?” For the secondary conviction outcome, respondents were asked, “Before you were age 26, were you ever convicted of a crime, including in juvenile court? Include a guilty plea as a conviction,” and then “How many times did this happen?” Although respondents could report both juvenile and adult arrests and convictions, distinguishing between juvenile and adult arrests and convictions was beyond the scope of this study. These measures, though, reflect the long-term impacts of ACEs and PCEs into young adulthood. Criminal behavior also peaks in adolescence and then declines, suggesting that by young adulthood (age 26 in our study) most individuals who will ever be involved in the criminal legal system will have already been involved (Sweeten et al., 2013). Using previously published methodology, we constructed the following primary and secondary outcome variables: (a) any arrest before age 26 (primary dichotomous outcome), (b) number of times arrested (count), (c) any conviction before age 26 (dichotomous), and (d) number of times convicted before age 26 years (count) (Barnert et al., 2023).

### Independent Variables-ACE and PCE Measures by Type

We used previously published methodology to construct the ACE and PCE measures by type from the PSID-CRCS, which asked respondents to retrospectively report their childhood experiences before age 17 (La Charite et al., 2023; Schickedanz et al., 2018). We mapped the PSID-CRCS survey items onto nine existing standard ACE questionnaire categories (Felitti et al., 1998; Schickedanz et al., 2018). The PSID-CRCS did not have questions that asked about parental incarceration or physical neglect. An affirmative response to any of the PSID-CRCS items that mapped to a single adverse experience category was coded as indicating that the individual had been exposed to that adverse experience. We created separate indicator variables for each adverse experience in the dataset that mapped to the original ACE framework: (i) physical, (ii) emotional, or (iii) sexual abuse; (iv) emotional neglect; (v) witnessing household intimate partner violence or (vi) parental substance use; (vii) having a parent with a mental health problem; (viii) parental separation or divorce; and (ix) having a deceased/estranged parent (Felitti et al., 1998; Felitti, 2002; Schickedanz et al., 2018). We summed the individual indicator variables to create an ACE count ranging from 0 to 9, which was then binned into standard categories (0, 1, 2–3, or 4 or more ACEs) (Schickedanz et al., 2018).

We constructed the PCE measures by type from the PSID-CRCS, which asked respondents about their positive experiences before age 17 for parent-related questions and from age 6 to 16 for school and neighborhood-related questions (La Charite et al., 2023). Twenty-six PSID-CRCS questions were identified that mapped onto items in existing PCE frameworks, including the PCE scale, the Benevolent Childhood Experiences (BCE) scale, and the Health Outcome from Positive Experience (HOPE) framework (Bethell et al., 2019; Guo et al., 2021; Narayan et al., 2018). We elected to include all 26 PSID-CRCS questions due to their high internal consistency (Cronbach’s alpha = 0.88). None of the PSID-CRCS Likert scale questions related to protective factors were excluded; however, the PSID-CRCS did not ask about all types of protective factors (e.g., supportive adult outside the home). Since we were constructing a PCE measure ad hoc and the PSID-CRCS questions did not perfectly align with any one specific framework, we utilized results from a separate factor analysis study of the 26 PSID-CRCS items, which identified five distinct types of PCEs: (i) healthy school climate and supportive peer relationships (e.g., happy at school, school safety, group of friends that you feel comfortable spending time with), (ii) neighborhood safety (e.g., safe to be out alone in my neighborhood during the day; at night; safe for children at night), (iii) neighborhood support (e.g., neighbors help each other, close knit), and nurturing (iv) paternal and (v) maternal relationships (e.g., communication, confide in, understand you, emotionally close, relationship quality) (La Charite et al., 2023). We separated the questions about maternal and paternal relationships into distinct categories based on a previously conducted factor analysis, suggesting that respondents perceived their relationships with each parent differently enough that it was not meaningful to combine these responses (La Charite et al., 2023). The PSID-CRCS questions, organized within these five PCE types, were associated with adult health outcomes (La Charite et al., 2023).

To create a PCE count score, we constructed five indicator variables for each of the five PCE types. The PSID-CRCS source item responses ranged from 1 (‘A lot’) to 4 (‘Never’). We reverse-coded and averaged the values of multiple survey source items within each of the five PCE types, so that a higher average indicated greater reported exposure to that PCE type. For the maternal and paternal relationship domains, if the respondent did not identify a primary maternal or paternal figure, they were assigned a value of ‘missing’ for those items. The five PCE type averages were then dichotomized separately at the 75th percentile, based on published methodology, as there are no standardized cut-off thresholds for the PSID-CRCS items (Guo et al., 2021). An average above the 75th percentile was coded as affirmative for strong exposure to the given PCE type. The individual five indicator variables were summed to create a PCE count ranging from 0 to 5, which was then binned into categories (0–1, 2–3, and 4–5 PCEs) (Barnert et al., 2023).

The PSID-CRCS survey items used to construct the ACE/PCE measures are published elsewhere (Barnert et al., 2023; La Charite et al., 2023; Schickedanz et al., 2018).

### Moderation by Sex

We hypothesized that the social construction of gender, rather than biological sex differences, may influence the association between different types of ACEs and PCEs and legal system outcomes. However, we will use the term sex differences as a proxy for gender due to a limitation that PSID only asked respondents for self- or proxy-reports of sex as male or female. We created a dichotomous variable for reported sex.

### Stratification by Race

Since different forms of racism could modify the association between ACE/PCE types and legal system outcomes, we constructed a race variable for stratified analysis (Lockwood et al., 2022; Novak, 2025). PSID obtained respondent self-reports and spouse/partner proxy reports on race. Response options included (1) White, (2) Black, African American, or Negro, (3) American Indian or Alaska Native, (4) Asian, (5) Native Hawaiian or Pacific Islander, (6) Other. Respondents could select multiple options. They were separately asked to self-report or proxy-report ethnicity as Spanish, Hispanic, or Latinx. For the stratified analysis, we generated a dichotomous variable for self or proxy-reported race/ethnicity as Non-Hispanic/Latinx White or Non-Hispanic/Latinx Black. The remaining racial/ethnic groups were excluded from the analytic sample for the stratified analysis. We divided race in this manner for the stratified analysis by race because of small counts for the other race and ethnic groups, which resulted in estimation problems. The ethnic groups were also too diverse to be combined into a single ‘Other’ category.

### Covariates-Demographics

We selected covariates that we believed were associated with ACEs, PCEs, and contact with the criminal legal system. Covariates included age in 2014 (continuous), highest educational attainment (less than high school, high school graduate or equivalent, college/vocational school/graduate school), household income (five-level categorical variable as a percent of the federal poverty limit), and number of individuals living within the household (continuous). We included sex and race/ethnicity as covariates when not testing for moderation by sex or stratifying by race. In this case, we constructed race and ethnicity as a four-category variable (Latinx/Hispanic, non-Hispanic/Latinx Asian/Pacific Islander, non-Hispanic/Latinx Black, non-Hispanic/Latinx White). Respondents in the American Indian/Alaskan Native and Other categories were excluded because their cells lacked sufficient respondents. Other than age, covariates were measured in 2013 when the respondent was over age 18. Since childhood household income information was not available in the data sources we analyzed, we used adult household income as a proxy since parental income and child earnings are correlated, and income is associated with ACEs and criminal legal system involvement (Behrman & Taubman, 1990; Shaefer et al., 2018; Tolliver et al., 2023).

## Statistical Analyses

### Bivariate Analyses

We used an adjusted Wald F test to assess differences in means across individuals arrested before age 26 for continuous variables, and we employed a design-based F test to examine associations between being arrested before age 26 and categorical variables.

### Multivariate Analyses

We used logistic (yes/no arrest or conviction) and negative binomial (count of arrests and convictions) regression to examine associations between ACEs, PCEs, and arrests and convictions before age 26.

### ACE Type Models

For the primary ACE type model, we included all nine ACE type indicator variables in the same model simultaneously while adjusting for the three PCE-count binned score categories (0–1, 2–3, 4–5 PCEs) and covariates. We elected to adjust for the PCE count rather than individual PCEs to reduce the number of variables in the model and collinearity. We calculated the predicted probabilities of the primary outcome for all nine ACE types.

### PCE Type Models

For the primary PCE type model, we included all five PCE type indicator variables in the same model simultaneously while adjusting for the four ACE-count binned score categories (0, 1, 2–3, 4 + ACEs) and covariates. We elected to adjust for the ACE-count, rather than individual ACEs, to reduce the number of variables in the model and collinearity. We calculated the predicted probabilities of the primary outcome for all five PCE types.

### Secondary Analysis by Sex and Race

We tested whether the association between exposure to childhood sexual abuse and arrest was modified by self-reported sex (as a proxy for gender) using moderation analysis followed by calculation of predicted probabilities. We focused on sexual abuse based on our a priori hypothesis that the link between this ACE type and legal system contact may differ by gender. In this case, because we tested only one type of ACE, we used moderation analysis to preserve power from the full sample.

For the sub-analysis by race, we stratified the primary ACE and PCE type models by the dichotomous variable of whether the respondent identified as Non-Hispanic/Latinx White or Non-Hispanic/Latinx Black. We elected to use stratification rather than moderation analysis for ease of interpretation, as we stratified across all nine ACE and five PCE types, since we did not have a priori hypotheses regarding which ACE and PCE types would be moderated by race. We limited our race-stratified analyses to arrests because, in our sample, convictions were far less common than arrests, resulting in cell sizes too small to support reliable or stable race-stratified models. In contrast, arrests occurred more frequently among young people, allowing analyses with adequate precision. Thus, to avoid producing uninterpretable or unstable estimates, we focused the race-stratified models on arrests.

### Missing

Based on our analytic sample definition, 11% of the original PSID-CRCS sample was dropped for the primary analysis. When we compared the socio-demographic characteristics of our analytic sample with the PSID-CRCS respondents who were excluded (*n* = 872), we found significant differences in age, race/ethnicity, education, household income, and household size. PSID-CRCS respondents excluded from the analytic sample were older, less educated, had lower incomes, smaller household sizes, and were more likely to identify as Black (Table 13). We adjusted for these factors in the regression models.

For the analytic sample, the amount of missing data for each ACE type ranged from 1% to 4%. The ACEs with the highest degree of missingness were parental divorce/separation (3%), parental substance use (3%), exposure to parental intimate partner violence (4%), and parental mental health problem (4%). The amount of missing data for each PCE type ranged from 1 to 2% and 10% due to the lack of an identified paternal figure. The amount of missing data for the arrest outcome was less than 1%.

Refer to the sensitivity analyses section below for details on how we tested an alternative analytic sample definition to assess the robustness of our findings under different assumptions about missing data.

### Sensitivity Analyses

We conducted seven sensitivity analyses to assess the robustness of the variable construction, including five for the ACE type model and two for the PCE type models.

For the ACE type models, we explored three alternative constructions for the PCE count variable since the PCE categories and counts are less established in the literature: a two-category PCE count (0–3 vs. 4–5 PCEs), a continuous PCE count, and a modified PCE count that excluded the neighborhood safety variable since it demonstrated a reverse association with arrests and convictions compared to the other PCE types. To address potential collinearity among ACE types, we tested nine separate models for each ACE type, adjusting for the continuous total ACE score minus the specific ACE of interest. Finally, to evaluate the robustness of our findings that excluded respondents who missed any of the ACE information, we evaluated an alternative sample definition that included respondents who partially completed some ACE and/or PCE items. We assigned a value of zero to missing ACE information as a conservative approach to assess the impact of missing data on our results.

For the PCE type model, we assessed two alternative sample definitions. First, similar to the ACE type model, we included respondents who completed some, but not all, of the ACE and PCE items using a revised analytic sample. Missing data on PCE types was assigned a value of zero. Second, we restricted the sample to respondents aged 18–58 in 2013, which included individuals who reached age 26 after 1980 (a crime control era) (Nellis, 2024; Thompson, 2010). This restriction was intended to ensure the generalizability of our findings to individuals who came of age during a time period focused on getting tough on crime. We also hypothesized that age and birth cohort might act as potential confounders, as younger respondents may have experienced (i) greater contact with the criminal justice system during the crime control era and (ii) birth cohort-related differences in childhood experiences.

We used Stata version 18.0 (StataCorp, College Station, TX) for all analyses (*New in Stata 18 | Stata*, 2024). Analyses were survey-weighted to account for complex survey sampling, address nonresponse, and achieve population representation. We used robust variance estimation to account for clustering within families. We did not perform a correction for multiple comparisons, as the outcomes (arrests, convictions) are highly correlated and the false discovery rate is therefore low. This analysis was approved by our institution’s Institutional Review Board (#16–000927). Funding sources were not involved in conducting or publishing this study.

## Results

The sample included 7,200 adults; 14.9% (weighted percent) reported being arrested (Table 1), and 5.9% reported being convicted before age 26. Among people arrested by age 26, the mean number of arrests was 2.1 (SD 4.2), and the mean number of convictions was 0.8 (SD 2.0). When comparing individuals who were and were not arrested before age 26, there were significant differences in exposure to all the ACE types except childhood emotional neglect and death/estrangement of a caregiver, as well as all PCE types except neighborhood safety (Table 1). Non-Hispanic/Latinx Black respondents disproportionally reported having experienced an arrest.

**Table 1:** Characteristics of eligible respondents in study sample, *N* = 7,200

	Overall No. (Weighted %)	Never arrested by age 26 No. (Weighted %)	Arrested by age 26 No. (Weighted %)	*p*-value^a^
	(*N* = 7,200)	*n* = 6,036 (85.1%)	*n* = 1,164 (14.9%)	
	48.5 (15.9)	49.5 (15.9)	42.8 (14.2)	
**Age in 2013, mean (SD)**				< 0.001
30–39 years	1,765 (19.9)	1,376 (78.9)	389 (21.1)	
40–49 years	1,227 (19.0)	1,037 (85.0)	190 (15.0)	
50–59 years	1,430 (21.5)	1,252 (86.0)	178 (14.0)	
60–69 years	1,017 (15.7)	922 (91.4)	95 (8.6)	
70–97 years	468 (10.2)	438 (94.3)	30 (5.7)	
**Sex**				< 0.001
Male	3,066 (47.0)	2,290 (76.9)	776 (23.1)	
Female	4,134 (53.0)	3,746 (92.4)	388 (7.6)	
**Race/Ethnicity**				0.006
Latinx	351 (6.1)	294 (85.0)	57 (15.0)	
Non-Latinx Asian/Pacific Islander	137 (2.5)	124 (91.6)	13 (8.4)	
Non-Latinx Black	1,895 (10.3)	1,516 (80.1)	379 (19.9)	
Non-Latinx White	4,723 (81.1)	4,022 (85.6)	701 (14.4)	
**Education**				< 0.001
Less Than High School	649 (7.9)	454 (73.4)	195 (26.6)	
High School Graduate or Equivalent	1,775 (24.8)	1,475 (84.2)	300 (15.8)	
College/Vocational/Graduate School	4,776 (67.3)	4,107 (86.8)	669 (13.2)	
**Household income**				< 0.001
<100% FPL	751 (7.9)	560 (72.7)	191 (27.3)	
100–199% FPL	1,052 (12.4)	839 (83.8)	213 (16.2)	
200–299% FPL	1,121 (15.4)	928 (84.8)	193 (15.2)	
300–400% FPL	976 (13.1)	820 (84.1)	156 (15.9)	
>400% FPL	3,300 (51.3)	2,889 (87.7)	411 (12.3)	
**Household size, mean (SD)**	2.5 (1.3)	2.5 (1.3)	2.6 (1.5)	0.16
** Number of ACEs**				< 0.001
0	2,401 (36.3)	2,138 (89.9)	263 (10.1)	
1	1,986 (26.7)	1,719 (86.3)	267 (13.7)	
2–3	2,014 (26.2)	1,589 (80.6)	425 (19.4)	
≥ 4	799 (10.7)	590 (76.7)	209 (23.3)	
**ACE Types**				
Emotional abuse	1,232 (16.6)	993 (81.3)	239 (18.7)	0.001
Physical abuse	1,658 (22.3)	1,277 (79.4)	381 (20.6)	< 0.001
Sexual abuse	275 (3.6)	193 (74.1)	82 (25.9)	< 0.001
Emotional neglect	519 (7.5)	411 (82.0)	108 (18.0)	0.09
Death or estrangement of caregiver(s)	350 (4.6)	276 (80.7)	74 (19.3)	0.06
Divorce or parental separation	1,963 (22.2)	1,517 (77.8)	446 (22.2)	< 0.001
Exposure to parental partner violence	1,492 (19.8)	1,164 (79.7)	328 (20.3)	< 0.001
Parent with a mental health problem	1,655 (24.2)	1,347 (82.3)	308 (17.7)	0.001
Exposure to parental substance use	1,396 (19.0)	1,056 (77.9)	340 (22.1)	< 0.001
**Number of PCEs**				< 0.001
0–3	6,257 (87.1)	5,188 (84.2)	1,069 (15.8)	
4–5	943 (12.9)	848 (91.4)	95 (8.6)	
**PCE Types**				
Neighborhood safety	4,577 (68.9)	3,919 (85.7)	658 (14.3)	0.11
Neighborhood support	3,263 (45.4)	2,803 (86.9)	460 (13.1)	0.002
Healthy school climate & supportive peers	2,209 (31.0)	1,925 (88.9)	284 (11.1)	< 0.001
Maternal relationship	1,590 (20.7)	1,375 (88.4)	215 (11.6)	0.001
Paternal relationship	1,633 (23.0)	1,406 (88.4)	227 (11.6)	0.002

^a^We used an adjusted Wald F test to test differences in means across arrested before age 26 for continuous variables; and we used a design-based F test to determine whether there were associations between arrested before age 26 and categorical variables

*ACE *adverse childhood experiences, *PCE *positive childhood experiences, *FPL *federal poverty limit, *SD *standard deviation

### Multivariate Analyses by ACE Type

#### Arrested before Age 26 by ACE Type (Table 2)

Controlling for PCE score and covariates, the adjusted odds of experiencing an arrest before age 26 was 1.70 (95% CI: [1.13, 2.57]) for sexual abuse, 1.55 (95% CI: [1.23, 1.96]) for parental substance use, 1.35 (95% CI: [1.11, 1.66]) for parental divorce, and 1.29 (95% CI: [1.04, 1.60]) for physical abuse compared to adults who did not report these individual exposures. We used the logistic regression estimates to calculate the predicted probabilities of an arrest after reporting different types of ACEs, providing a more interpretable measure of effect size than the odds ratio. We found that the likelihood of being arrested before age 26 was 21% with a history of sexual abuse, 19% for parental substance use, 17% for parental divorce/separation, and 17% for physical abuse.

**Table 2:** Adjusted odds ratios (OR) and predicted probabilities with 95% confidence intervals (CI) for logistic regression models of arrested before age 26 on ACE type with PCE count adjustment

	Arrested Before Age 26			
	Model with PCE (3 category) Adjustment		Model with PCE (2 category) Adjustment	Model with PCE (continuous) Adjustment
	*n* = 7,106		*n* = 7,106	*n* = 7,106
	Adjusted^a^ OR (95% CI)	Predicted Probability^b^ % (95% CI) for ACE type exposure vs. no exposure	Adjusted^a^ OR (95% CI)	Adjusted^a^ OR (95% CI)
**ACE Types**				
Emotional abuse	1.16 (0.91, 1.48)	16 (14, 19) vs. 15 (13, 16)	1.15 (0.91, 1.47)	1.15 (0.90, 1.46)
Physical abuse	1.29 (1.04, 1.60)*	17 (15, 19) vs. 14 (13, 15)	1.29 (1.04, 1.59)*	1.30 (1.05, 1.62)*
Sexual abuse	1.70 (1.13, 2.57)*	21 (16, 27) vs. 15 (13, 16)	1.69 (1.12, 2.55)*	1.69 (1.12, 2.56)*
Emotional neglect	0.96 (0.70, 1.32)	14 (11, 18) vs. 15 (14, 16)	0.95 (0.69, 1.31)	0.97 (0.70, 1.33)
Death or estrangement of caregiver(s)	1.32 (0.90, 1.93)	18 (13, 23) vs. 15 (14, 16)	1.30 (0.88, 1.91)	1.31 (0.89, 1.92)
Parental divorce/separation	1.35 (1.11, 1.66)**	17 (15, 20) vs. 14 (13, 15)	1.34 (1.10, 1.63)**	1.35 (1.11, 1.65)**
Exposure to parental partner violence	1.18 (0.94, 1.48)	16 (14, 19) vs. 14 (13, 16)	1.17 (0.94, 1.47)	1.18 (0.94, 1.48)
Parent with a mental health problem	1.08 (0.89, 1.33)	16 (14, 18) vs. 15 (13, 16)	1.07 (0.87, 1.31)	1.08 (0.88, 1.32)
Exposure to parental substance use	1.55 (1.23, 1.96)***	19 (16, 22) vs. 14 (13, 15)	1.55 (1.23, 1.95)***	1.55 (1.23, 1.96)***
**PCE count (3 category)**				
0–1	reference			
2–3	1.15 (0.95, 1.39)			
4–5	0.76 (0.56, 1.04)			
**PCE count (2 category)**				
0–3			reference	
4–5			0.71 (0.52, 0.95)*	
**PCE count (continuous)**				
0–5				0.99 (0.93, 1.06)

*p-value < 0.05; **p-value < 0.01; ***p-value < 0.001

^a^Estimates adjusted for age, sex, race, education, family income, and household size

^b^Predicted probabilities for each ACE type were generated by setting the other variables to their observed values

*ACE *adverse childhood experiences, *PCE *positive childhood experiences

#### Number of Arrests before Age 26 by ACE Type (Table 3)

The same ACE types were associated with the number of arrests before age 26. Among individuals who experienced an arrest before age 26, exposure to sexual abuse increased the expected number of arrests by a factor of 1.80 (95% CI [1.23, 2.62]), parental substance use by a factor of 1.55 (95% CI [1.22, 1.98]), parental divorce by a factor of 1.54 (95% CI: [1.16, 2.04]), and physical abuse by a factor of 1.43 (95% CI: [1.13, 1.80]), as compared to respondents without these exposures, after adjusting for PCE score and covariates.

**Table 3:** Adjusted incidence rate ratios (IRR) with 95% confidence intervals (CI) for negative binomial regression models of times arrested before age 26 on ACE type with adjustment of PCE count

	Times Arrested Before Age 26	Convicted Before Age 26	Times Convicted Before Age 26
	*n* = 7,097	*n* = 7,097	*n* = 7,092
	Adjusted^a^ IRR (95% CI)	Adjusted^a^ OR (95% CI)	Adjusted^a^ IRR (95% CI)
**ACE Types**			
Emotional abuse	1.33 (0.99, 1.80)	1.42 (1.04, 1.93)*	1.47 (1.04, 2.08)*
Physical abuse	1.43 (1.13, 1.80)**	1.54 (1.13, 2.08)**	2.07 (1.48, 2.89)***
Sexual abuse	1.80 (1.23, 2.62)**	1.58 (0.90, 2.77)	1.57 (0.86, 2.86)
Emotional neglect	1.29 (0.78, 2.14)	0.76 (0.47, 1.21)	1.57 (0.78, 3.16)
Death or estrangement of caregiver(s)	0.95 (0.64, 1.40)	1.60 (0.96, 2.67)	1.29 (0.74, 2.25)
Parental divorce/separation	1.54 (1.16, 2.04)**	1.63 (1.23, 2.16)**	1.55 (1.14, 2.11)**
Exposure to parental partner violence	1.03 (0.81, 1.30)	1.08 (0.79, 1.48)	0.88 (0.64, 1.20)
Parent with a mental health problem	1.06 (0.84, 1.35)	1.41 (1.04, 1.90)*	1.69 (1.22, 2.35)**
Exposure to parental substance use	1.55 (1.22, 1.98)***	1.33 (0.96, 1.85)	1.27 (0.90, 1.79)
**PCE Count**			
0–1	reference	reference	reference
2–3	1.19 (0.96, 1.48)	1.29 (0.98, 1.71)	1.33 (0.99, 1.80)
4–5	0.82 (0.58, 1.16)	1.06 (0.66, 1.71)	1.11 (0.68, 1.80)

*p-value < 0.05; **p-value < 0.01; ***p-value < 0.001

^a^Estimates adjusted for age, sex, race, education, family income, and household size

*ACE *adverse childhood experiences, *PCE *positive childhood experiences

Same models were also tested with two category PCE count (0–3, 4–5) with consistent results

#### Convicted before Age 26 by ACE Type (Table 3)

In addition to being associated with arrests and arrest counts, parental divorce and physical abuse were also associated with convictions. Parental substance use and sexual abuse were correlated with arrests, but not convictions. Emotional abuse and parental mental health problems were newly associated with convictions. Controlling for PCE score and covariates, the adjusted odds of experiencing a conviction before age 26 was 1.63 (95% CI: [1.23, 2.16]) for parental divorce, 1.54 (95% CI: [1.13, 2.08]) physical abuse, 1.42 (95% CI: [1.04, 1.93]) for emotional abuse, and 1.41 (95% CI: [1.04, 1.90]) for parental mental health problem compared to respondents without these exposures.

#### Times Convicted before Age by ACE Type (Table 3)

The same four ACE types were correlated with both convictions and the total number of convictions. Parental divorce and physical abuse were associated with the number of convictions, in addition to being correlated with arrests, arrest counts, and convictions. Besides being associated with convictions, parental mental health problems and emotional abuse were also associated with the conviction count. Although associated with arrests, parental substance use, and sexual abuse were not associated with the conviction count. Among individuals who experienced a conviction, exposure to physical abuse increased the expected number of convictions by a factor of 2.07 (95% CI: [1.48, 2.89]), parental mental health problem by a factor of 1.69 (95% CI: [1.22, 2.35]), parental divorce by a factor of 1.55 (95% CI: [1.14, 2.11]), and emotional abuse by a factor of 1.47 (95% CI: [1.04, 2.08]) as compared to respondents without these exposures, after covariate and PCE score adjustment.

#### PCE Count Sources (Tables 2 & 3)

Reporting greater PCE exposure using the 3-category PCE score was associated with lower odds of arrest, but the association was not significant (aOR 0.76, 95% CI: 0.56–1.04), after adjusting for covariates and ACE types. The three-category PCE score was also not significantly associated with the number of arrests, convictions, or times convicted, after adjusting for covariates and ACE types.

### Multivariate Analyses by PCE Type

#### Arrested before Age 26 by PCE Type (Table 4)

After controlling for ACE score and covariates, reporting perception of greater neighborhood safety in childhood was associated with increased odds of being arrested before age 26 (aOR 1.27, 95% CI: [1.02,1.58]).

**Table 4:** Adjusted odds ratios (OR) and predicted probabilities with 95% confidence intervals (CI) for logistic regression models of arrested before age 26 on PCE type with ACE count adjustment

	Arrested Before Age 26		
	Model with ACE (4 category) Adjustment	Model with ACE (4 category) Adjustment	Model with ACE (continuous) Adjustment
	*n* = 6,369	*n* = 6,369	*n* = 6,369
	Adjusted^a^ OR (95% CI)	Predicted Probability^b^ % (95% CI) for high vs. low PCE type exposure	Adjusted^a^ OR (95% CI)
**PCE Types**			
Neighborhood safety	1.27 (1.02, 1.58)*	15 (14, 17) vs. 13 (11, 15)	1.28 (1.02, 1.60)*
Neighborhood support	0.99 (0.80, 1.23)	14 (13, 16) vs. 15 (13, 16)	1.00 (0.80, 1.23)
Healthy school climate and peer support	1.01 (0.82, 1.25)	15 (13, 17) vs. 14 (13, 16)	1.01 (0.82, 1.24)
Nurturing maternal relationship	0.85 (0.67, 1.07)	13 (11, 15) vs. 15 (14, 16)	0.86 (0.68, 1.08)
Nurturing paternal relationship	0.80 (0.64, 1.01)	13 (11, 15) vs. 15 (14, 16)	0.81 (0.65, 1.01)
**ACE count (categorical)**			
0	reference		
1	1.42 (1.10, 1.83)**		
2–3	1.99 (1.57, 2.52)***		
≥ 4	2.68 (2.01, 3.57)***		
**ACE count (continuous)**			
0–9			1.25 (1.18, 1.31)***

*p-value < 0.05; **p-value < 0.01; ***p-value < 0.001

^a^Estimates adjusted for age, sex, race, education, family income, and household size

^b^Predicted probabilities for each PCE type were generated by setting the other variables to their observed values

*ACE *adverse childhood experiences, *PCE *positive childhood experiences

#### Number of Arrests before Age 26 by PCE Type (Table 5) 

Higher reported exposure to a nurturing paternal relationship – but not a nurturing maternal relationship – was associated with a decrease in the expected number of arrests by a factor of 0.73 (95% CI: [0.55, 0.99]) after adjusting for ACE score and covariates. After adjusting for ACE score and covariates, participants’ reports of a high level of neighborhood safety in childhood were associated with an increase in the expected number of arrests by a factor of 1.30 (95% CI: [1.00, 1.69]).

**Table 5:** Adjusted odds ratios (OR) and incidence rate ratios (IRR) with 95% confidence intervals (CI) for Binomial regression models of times arrested and convicted and logistic regression model of convicted on PCE type with adjustment of ACE count

	Times Arrested Before Age 26	Convicted Before Age 26	Times Convicted Before Age 26
	*n* = 6,362	*n* = 6,360	*n* = 6,356
	Adjusted^a^ IRR (95% CI)	Adjusted^a^ OR (95% CI)	Adjusted^a^ IRR (95% CI)
**PCE Types**			
Neighborhood safety	1.30 (1.00, 1.69)*	1.52 (1.11, 2.09)**	1.66 (1.14, 2.42)**
Neighborhood support	0.87 (0.70, 1.09)	0.92 (0.67, 1.27)	0.84 (0.60, 1.18)
Healthy school climate and peer support	0.97 (0.78, 1.21)	0.88 (0.63, 1.24)	0.78 (0.53, 1.14)
Nurturing maternal relationship	1.12 (0.77, 1.64)	1.00 (0.72, 1.39)	1.63 (0.97, 2.75)
Nurturing paternal relationship	0.73 (0.55, 0.99)*	0.91 (0.66, 1.27)	0.77 (0.51, 1.15)
**ACE Count**			
0	reference	reference	reference
1	1.34 (1.05, 1.71)*	1.75 (1.17, 2.61)**	1.76 (1.19, 2.60)**
2–3	2.38 (1.82, 3.11)***	2.64 (1.84, 3.80)***	3.53 (2.34, 5.32)***
≥ 4	3.14 (2.33, 4.24)***	4.11 (2.68, 6.32)***	6.27 (4.00, 9.83)***

*p-value < 0.05; **p-value < 0.01; ***p-value < 0.001

^a^Estimates adjusted for age, sex, race, education, family income, and household size

*ACE *adverse childhood experiences, *PCE *positive childhood experiences

#### Convicted before Age 26 by PCE Type (Table 5)

Controlling for ACE score and covariates, the adjusted odds of experiencing a conviction before age 26 were 1.52 (95% CI: [1.11, 2.09]) for individuals who reported high neighborhood safety in childhood, compared to those with low exposure.

#### Times Convicted before Age 26 by PCE Type (Table 5)

Among individuals who experienced a conviction before age 26, participant report of a high level of neighborhood safety in childhood was associated with an increase in the expected number of convictions by a factor of 1.66 (95% CI: [1.14, 2.42]) after ACE score and covariate adjustment.

#### ACE Count Score (Table 4 & 5)

There was a significant dose-response relationship between the level of ACE exposure and all four legal system outcomes.

### Arrested before Age 16 by Race and Sex

#### ACEs and Arrested before Age 26 by Race (Table 6)

The individual associations between exposure to parental divorce and arrest and between parental substance use and arrest remained significant for both racial groups (Non-Hispanic/Latinx White and Non-Hispanic/Latinx Black), but the adjusted odds ratios were higher for individuals who identified as Black. The other previously significant associations between exposure to sexual abuse and arrest and physical abuse and arrest were only significant for individuals who identified as Black. Conversely, exposure to a parent with a mental health problem was associated with a reduced odds of being arrested before age 26 (aOR 0.51, 95% CI: [0.27, 0.96]) for individuals who identified as Black. The reported analyses were adjusted for covariates and PCE score.

**Table 6:** Adjusted odds ratios (OR) with 95% confidence intervals (CI) for logistic regression models of arrested before age 26 on ACE type, stratified by race

	Arrested Before Age 26	
	Non-Hispanic/Latinx White	Non-Hispanic/Latinx Black
	*n* = 4,723	*n* = 1,895
	Adjusted^a^ OR (95% CI)	Adjusted^a^ OR (95% CI)
**ACE Types**		
Emotional abuse	1.22 (0.93, 1.61)	0.93 (0.52, 1.64)
Physical abuse	1.26 (0.98, 1.61)	2.11 (1.35, 3.29)**
Sexual abuse	1.42 (0.87, 2.30)	3.16 (1.24, 8.06)*
Emotional neglect	0.98 (0.69, 1.40)	0.48 (0.22, 1.07)
Death or estrangement of caregiver(s)	1.34 (0.85, 2.09)	1.03 (0.38, 2.83)
Parental divorce/separation	1.32 (1.03, 1.68)*	1.69 (1.09, 2.61)*
Exposure to parental partner violence	1.14 (0.88, 1.47)	1.61 (0.95, 2.72)
Parent with a mental health problem	1.17 (0.93, 1.46)	0.51 (0.27, 0.96)*
Exposure to parental substance use	1.34 (1.03, 1.74)*	2.70 (1.65, 4.40)***
**PCE count (3 category)**		
0–1	reference	reference
2–3	1.17 (0.95, 1.44)	0.99 (0.61, 1.60)
4–5	0.59 (0.41, 0.86)**	1.22 (0.64, 2.31)

*p-value < 0.05; **p-value < 0.01; ***p-value < 0.001

^a^Estimates adjusted for age, sex, race, education, family income, and household size

*ACE *adverse childhood experiences, *PCE *positive childhood experiences

In the same model, reporting 4–5 PCEs was significantly associated with a lower odds of arrest for individuals who identified as Non-Hispanic/Latinx White (aOR 0.59, 95% CI: 0.41–0.86), but not for individuals who identified as Non-Hispanic/Latinx Black. The reported analyses were adjusted for covariates and ACE score.

#### PCEs and Arrested before Age 26 by Race (Table 7)

Greater exposure to nurturing maternal (aOR 0.73, 95% CI: [0.55, 0.96]) and paternal (aOR 0.75, 95% CI: [0.57, 0.97]) relationships were associated with a decrease in the odds of being arrested before age 26 for individuals who identified as Non-Hispanic/Latinx White. In contrast, greater reported perceived childhood neighborhood safety was associated with higher odds of reporting arrest before age 26 (aOR 1.39, 95% CI: [1.08, 1.79]) only for individuals who identified as Non-Hispanic/Latinx White. None of the PCE type associations were significant for individuals who identified as Non-Hispanic/Latinx Black.

**Table 7:** Adjusted odds ratios (OR) with 95% confidence intervals (CI) for logistic regression models of arrested before age 26 on PCE type, stratified by race

	Arrested Before Age 26	
	Non-Hispanic/Latinx White	Non-Hispanic/Latinx Black
	*n* = 4,448	*n* = 1,491
	Adjusted^a^ OR (95% CI)	Adjusted^a^ OR (95% CI)
**PCE Types**		
Neighborhood safety	1.39 (1.08, 1.79)*	0.67 (0.39, 1.18)
Neighborhood support	0.97 (0.77, 1.22)	1.25 (0.63, 2.48)
Healthy school climate and peer support	1.04 (0.83, 1.31)	1.35 (0.72, 2.52)
Nurturing maternal relationship	0.73 (0.55, 0.96)*	1.10 (0.70, 1.73)
Nurturing paternal relationship	0.75 (0.57, 0.97)*	1.22 (0.73, 2.06)
**ACE count**		
0	reference	reference
1	1.42 (1.08, 1.87)*	1.01 (0.47, 2.20)
2–3	2.02 (1.55, 2.62)***	2.64 (1.28, 5.43)**
≥ 4	2.35 (1.69, 3.27)***	4.13 (1.87, 9.14)***

*p-value < 0.05; **p-value < 0.01; ***p-value < 0.001

^a^Estimates adjusted for age, sex, race, education, family income, and household size

*ACE *adverse childhood experiences, *PCE *positive childhood experiences

For the same models, the ACE score was significantly associated with arrests in each case except reporting one ACE compared to zero ACEs for the group that identified as Non-Hispanic/Latinx Black. Before stratification, all levels of the ACE score were significantly associated with arrest.

#### Sexual Abuse and Arrested before Age 26 by Sex (Fig. 2)

Using the overall test of interaction, the relationship between arrests and sexual abuse was different for females and males (*p* < 0.05). There was a significant association between sexual abuse and arrest only for females, controlling for covariates and PCE score. The predicted probability of being arrested before age 26 was 7% for females who did not experience sexual abuse as a child, compared to 15% for females who did experience sexual abuse as a child. A similar pattern was seen for the secondary convictions and the number of conviction outcomes (results not shown).

**Fig. 2:**
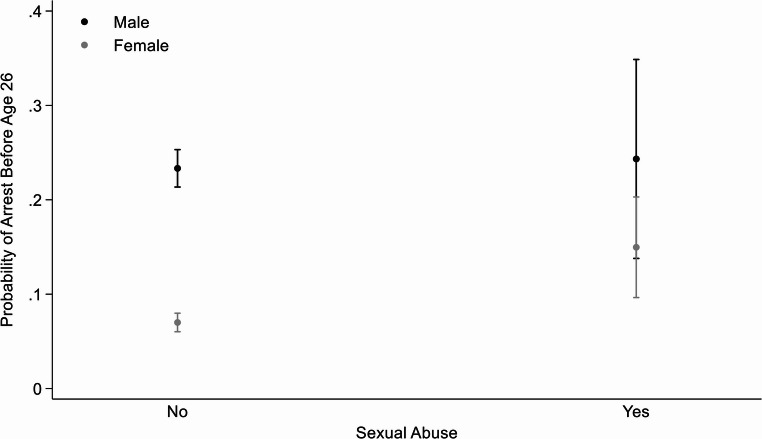
Predicted probability of being arrested before age 26 based on exposure to childhood sexual abuse, stratified by sex

### Sensitivity Analyses (Tables 2, 8, 9, 10, 11, and 12)

When we adjusted for the total ACE score minus the specific ACE type rather than each individual type of ACEs, we lost significance for the association between childhood emotional abuse and conviction before age 26 (Table 8). However, the direction of the association was consistent, and the significance returned for the times convicted outcome.

**Table 8:** Adjusted odds ratios (OR) and incidence rate ratios (IRR) with 95% confidence intervals (CI) for logistic regression models of arrested and negative binomial regression models of times arrested before age 26 on ACE type with adjustment for total ACE score minus specific ACE Type

	Arrested Before Age 26	Times Arrested Before Age 26	Convicted Before Age 26	Times Convicted Before Age 26
	*n* = 7,106	*n* = 7,097	*n* = 7,097	*n* = 7,092
**ACE Types**	Adjusted^a^ OR (95% CI)	Adjusted^a^ IRR (95% CI)	Adjusted^a^ OR (95% CI)	Adjusted^a^ IRR (95% CI)
Emotional abuse	1.10 (0.87, 1.40)	1.31 (0.91, 1.87)	1.30 (0.96, 1.78)	1.65 (1.01, 2.70)*
Physical abuse	1.25 (1.01, 1.55)*	1.37 (1.04, 1.80)*	1.45 (1.08, 1.96)*	2.03 (1.35, 3.06)**
Sexual abuse	1.66 (1.11, 2.49)*	1.78 (1.21, 2.63)**	1.61 (0.93, 2.79)	1.69 (0.88, 3.21)
Emotional neglect	0.95 (0.69, 1.29)	1.29 (0.73, 2.31)	0.77 (0.49, 1.23)	1.71 (0.70, 4.20)
Death or estrangement of caregiver(s)	1.31 (0.89, 1.92)	0.97 (0.66, 1.43)	1.56 (0.93, 2.61)	1.24 (0.70, 2.19)
Parental divorce/separation	1.36 (1.11, 1.67)**	1.54 (1.16, 2.06)**	1.62 (1.22, 2.14)**	1.54 (1.11, 2.13)**
Exposure to parental partner violence	1.21 (0.98, 1.51)	1.05 (0.81, 1.36)	1.08 (0.80, 1.47)	0.85 (0.61, 1.20)
Parent with a mental health problem	1.12 (0.92, 1.37)	1.10 (0.86, 1.40)	1.43 (1.06, 1.92)*	1.65 (1.15, 2.36)**
Exposure to parental substance use	1.53 (1.21, 1.93)***	1.43 (1.09, 1.87)*	1.28 (0.92, 1.78)	1.08 (0.73, 1.59)

*p-value < 0.05; **p-value < 0.01; ***p-value < 0.001

^a^Estimates adjusted for three-category PCE score, age, sex, race, education, family income, household size.

^b^For example, for emotional abuse, the model adjusted for the total ACE score minus emotional abuse (The ACE score summed across physical & sexual abuse, neglect, death, divorce, partner violence, mental health problem, substance use – but not emotional abuse)

*ACE *adverse childhood experiences, *PCE *positive childhood experiences

We gained additional power to detect that higher PCE exposure was significantly associated with lower odds of arrest using the two-category PCE count binned score (Table 2), the two and three category PCE count without neighborhood safety (Table 9), and the two and three category PCE count when the missing ACE/PCE items were set to zero to retain respondents in the analytic sample (Table 10), but not with the continuous PCE count (Table 2). When we set missing ACE/PCE items to zero to retain respondents in the analytic sample and when we restricted the age range to 18–58 to stratify by birth year, we lost significance for neighborhood safety and gained significance for nurturing paternal relationship (Tables 11 and 12), suggesting that greater exposure to nurturing paternal relationship is associated with reduced likelihood of arrest. The remaining sensitivity analyses were similar to the primary results.

**Table 9:** Adjusted odds ratios (OR) with 95% confidence intervals (CI) for logistic regression models of arrested before age 26 on ACE type with adjustment of PCE count minus the neighborhood safety PCE type

	Arrested Before Age 26	
	Model with 3 Category PCE Count	Model with 2 Category PCE Count
	*n* = 7,106	*n* = 7,106
	Adjusted^a^ OR (95% CI)	Adjusted^a^ OR (95% CI)
**ACE Types**		
Emotional abuse	1.15 (0.90, 1.46)	1.15 (0.90, 1.47)
Physical abuse	1.29 (1.04, 1.60)*	1.29 (1.04, 1.60)*
Sexual abuse	1.69 (1.12, 2.54)*	1.69 (1.12, 2.55)*
Emotional neglect	0.96 (0.70, 1.32)	0.96 (0.70, 1.32)
Death/estrangement of caregiver(s)	1.31 (0.89, 1.92)	1.31 (0.89, 1.93)
Parental divorce/separation	1.34 (1.10, 1.63)**	1.34 (1.10, 1.64)**
Exposure to parental partner violence	1.17 (0.94, 1.47)	1.17 (0.94, 1.47)
Parent with a mental health problem	1.07 (0.88, 1.31)	1.08 (0.88, 1.32)
Exposure to parental substance use	1.54 (1.22, 1.94)***	1.54 (1.22, 1.94)***
**PCE count (3 category)**		
0–1	reference	
2–3	0.96 (0.79, 1.16)	
4	0.44 (0.24, 0.82)*	
**PCE count (2 category)**		
0–3		reference
4		0.45 (0.24, 0.83)*

*p-value < 0.05; **p-value < 0.01; ***p-value < 0.001

^a^Estimates adjusted for age, sex, race, education, family income, and household size

*ACE *adverse childhood experiences, *PCE *positive childhood experiences

**Table 10:** Adjusted odds ratios (OR) with 95% confidence intervals (CI) for logistic regression models of arrested before age 26 on ACE type with adjustment of PCE count, partial responses (incomplete cases) to ACE/PCE items included in analytic sample

	Arrested Before Age 26	
	Model with 3 Category PCE Count	Model with 2 Category PCE Count
	*n* = 7,885	*n* = 7,885
	Adjusted^a^ OR (95% CI)	Adjusted^a^ OR (95% CI)
**ACE Types**		
Emotional abuse	1.10 (0.87, 1.40)	1.10 (0.87, 1.39)
Physical abuse	1.33 (1.08, 1.63)**	1.32 (1.08, 1.63)**
Sexual abuse	1.65 (1.11, 2.45)*	1.64 (1.10, 2.44)*
Emotional neglect	1.01 (0.75, 1.37)	1.00 (0.74, 1.35)
Death or estrangement of caregiver(s)	1.37 (0.96, 1.97)	1.36 (0.95, 1.95)
Parental divorce/separation	1.35 (1.11, 1.63)**	1.34 (1.10, 1.62)**
Exposure to parental partner violence	1.21 (0.97, 1.50)	1.20 (0.97, 1.49)
Parent with a mental health problem	1.10 (0.91, 1.33)	1.09 (0.90, 1.32)
Exposure to parental substance use	1.58 (1.27, 1.97)***	1.58 (1.27, 1.97)***
**PCE count (3 category)**		
0–1	Reference	
2–3	1.10 (0.92, 1.31)	
4–5	0.73 (0.55, 0.99)*	
**PCE count (2 category)**		
0–3		reference
4–5		0.70 (0.52, 0.93)*

*p-value < 0.05; **p-value < 0.01; ***p-value < 0.001

^a^Estimates adjusted for age, sex, race, education, family income, and household size

*ACE *adverse childhood experiences, *PCE *positive childhood experiences

**Table 11:** Adjusted odds ratios (OR) with 95% confidence intervals (CI) for logistic regression model of arrested before age 26 on PCE type with adjustment of ACE count, partial responses (incomplete cases) to ACE/PCE items included in analytic sample

	Arrested Before Age 26
	*n* = 7,885
	Adjusted^a^ OR (95% CI)
**PCE Types**	
Neighborhood safety	1.22 (1.00, 1.51)
Neighborhood support	0.97 (0.80, 1.19)
Healthy school climate and peer support	0.97 (0.80, 1.17)
Nurturing maternal relationship	0.86 (0.69, 1.06)
Nurturing paternal relationship	0.78 (0.64, 0.97)*
**ACE count**	
0	reference
1	1.43 (1.14, 1.81)**
2–3	2.08 (1.67, 2.59)***
≥ 4	2.86 (2.17, 3.76)***

*p-value < 0.05; **p-value < 0.01; ***p-value < 0.001

^a^Estimates adjusted for age, sex, race, education, family income, and household size

*ACE *adverse childhood experiences, *PCE *positive childhood experiences

**Table 12:** Adjusted odds ratios (OR) with 95% confidence intervals (CI) for logistic regression model of arrested before age 26 on PCE type with adjustment by ACE count, sample restricted to respondents ages 18–58

	Arrested Before Age 26
	*n* = 4,902
	Adjusted^a^ OR (95% CI)
P**CE Types**	
Neighborhood safety	1.26 (1.00, 1.59)
Neighborhood support	1.02 (0.81, 1.28)
Healthy school climate and peer support	0.96 (0.76, 1.22)
Nurturing maternal relationship	0.89 (0.69, 1.15)
Nurturing paternal relationship	0.77 (0.61, 0.97)*
**ACE count**	
0	reference
1	1.37 (1.04, 1.80)*
2–3	1.96 (1.51, 2.54)***
≥ 4	2.52 (1.86, 3.42)***

*p-value < 0.05; **p-value < 0.01; ***p-value < 0.001

^a^Estimates adjusted for age, sex, race, education, family income, and household size

*ACE *adverse childhood experiences, *PCE *positive childhood experiences

**Table 13:** Demographic comparisons between the analytic sample and PISD-CRCS respondents who were excluded from the analytic sample

	Analytic Sample	Excluded Sample	*p*-value
	No. (Weighted %)	No. (Weighted %)	
	*n* = 7200	*n* = 872	
**Age in years, mean (SD)**	48.5 (15.9)	58.4 (17.8)	< 0.001
**Sex**			0.34
Male	3,066 (47.0)	355 (44.9)	
Female	4,134 (53.0)	517 (55.1)	
**Race/Ethnicity**			< 0.00
Non-Latinx Asian	137 (2.5)	17 (2.4)	
Non-Latinx Black	1,895 (10.3)	381 (19.0)	
Hispanic/Latino	351 (6.1)	37 (5.0)	
Non-Latinx White	4,723 (81.1)	423 (73.6)	
**Education**			< 0.00
Less Than High School	649 (7.9)	202 (20.8)	
High School Graduate or Equivalent	1,775 (24.8)	306 (33.2)	
College/Vocational/Graduate School	4,776 (67.3)	364 (46.0)	
**Household Income**			< 0.00
<100% FPL	751 (7.9)	177 (13.9)	
100–199% FPL	1,052 (12.4)	193 (19.7)	
200–299% FPL	1,121 (15.4)	151 (18.0)	
300–400% FPL	976 (13.1)	96 (12.5)	
>400% FPL	3,300 (51.3)	255 (35.8)	
**Household size, mean (SD)**	2.5 (1.3)	2.1 (1.3)	< 0.00

*PSID-CRCS* panel study of income dynamics – childhood retrospective circumstance study, *FPL* federal poverty level adjusted wald and pearson tests were used to compare the demographic characteristics between the analytic sample and PSID-CRCS respondents who were excluded from the analytic sample

## Discussion

The present study is among the first that we know of to use U.S. nationally representative data to examine how individual types of ACEs and PCEs are related to the likelihood and number of arrests and convictions by young adulthood (i.e., by age 26). Among ACE types, childhood sexual abuse, parental substance use, parental divorce/separation, and physical abuse demonstrated the strongest associations with *arrests* and the *number of arrests* occurring before or during young adulthood. Parental divorce/separation, physical abuse, emotional abuse, and a parent with a mental health problem were strongly linked to *convictions* and *number of convictions*. Accordingly, parental divorce/separation and physical abuse were the only ACE types that maintained significance from arrest to conviction. By PCE type, a nurturing paternal relationship was significantly associated with fewer arrests, but other individual PCE types were not independently protective against arrests or convictions in the entire sample. These findings suggest that while certain ACEs mark heightened risk for early legal system involvement, no individual PCE type consistently emerged as independently protective.

The relationship between overall PCE exposure (PCE score) and arrest varied depending on how the score was constructed. The three-category and continuous PCE scores were not significantly associated with arrest. However, collapsing the PCE score into two categories or setting missing ACE/PCE data to zero, thereby retaining a larger analytic sample, revealed that reporting four or more PCEs was associated with significantly lower odds of arrest, suggesting that the initial construction lacked sufficient power and may indicate a threshold effect. A significantly protective association was again identified when neighborhood safety was excluded from the PCE score, indicating that the neighborhood safety domain may be driving the initial null relationship.

The secondary analyses by race/ethnicity and gender revealed further nuance. The number of associations between ACE types and arrests, and the degree of the associations, was stronger for individuals identifying as Non-Hispanic/Latinx Black compared to Non-Hispanic/Latinx White. Greater PCE exposure - specifically nurturing parental relationships - was protective against arrests for Non-Hispanic/Latinx White individuals but not for Non-Hispanic/Latinx Black respondents. Experiencing sexual abuse was identified as a risk factor for arrest among females but not males. Future research can explore whether there are gender differences in the associations between other types of ACEs and PCEs and legal system outcomes.

Unexpectedly, childhood perceptions of greater neighborhood safety were also associated with higher odds of arrest and conviction, particularly for non-Hispanic White youth. We initially hypothesized that this finding may have occurred due to cohort effects contributing to both reduced perceived neighborhood safety and increased policing and arrests over time due to policy changes that led to the focus on drug-related crimes and the rise of the crime control era (Thompson, 2010). More specifically, there may be racial differences in perceptions of neighborhood safety (Jordan & Gabbidon, 2010) or there could be ceiling effects – Black youth may already face systematically higher baseline risk of contact with the criminal legal system compared to White youth, so that perceptions of neighborhood safety have less of an impact (Abrams et al., 2021). However, the neighborhood safety association was diminished in a sensitivity analysis that accounted for birth cohorts during the crime control era and was not observed in the bivariate model. Therefore, we caution readers against over-interpreting this result. Further study is warranted to understand the associations between perceived neighborhood safety in childhood, changing patterns in neighborhood policing presence and practices, and risk of arrests and convictions, as well as to test item performance across birth cohorts.

### Comparison to Existing Literature

Our ACE findings are consistent with prior studies demonstrating significant associations between child abuse, parental separation or divorce, and parental substance use with criminal legal system outcomes among U.S. youth (Barnert et al., 2021; Basto-Pereira et al., 2016; Brummer et al., 2021; King et al., 2009). This study extends prior research by identifying the most consequential ACE types and describing their relative contribution to a young person’s risk of arrest and convictions while adjusting for other types of ACEs and PCE exposure. For our PCE findings, other studies have likewise found that greater overall PCE exposure was associated with improved criminal legal system outcomes, including less recidivism (Baglivio & Wolff, 2021). As a potential mechanism, another study found that greater PCE exposure counteracted the negative influence of ACEs by improving self-control among youth on probation (Mueller & Carey, 2022).

The strongest associations between ACE and PCE types and arrests or convictions observed in this study also differed from those previously reported for health outcomes. In these prior health outcome studies, a larger number of ACE types were independently associated with depression and post-traumatic stress disorder, ACE type patterns varied for health behaviors and chronic disease outcomes, and peer support and healthy school climate was the PCE type most robustly associated with physical and mental health outcomes (Chang et al., 2019; La Charite et al., 2023). This differs from studies using composite measures of ACEs and PCEs that show similar graded relationships between ACE and PCE scores and legal system and health outcomes (Baglivio & Wolff, 2021; Fagan & Novak, 2018; Felitti et al., 1998; Huang et al., 2023). Future studies can further explore whether ACE and PCE risk assessment and intervention may require the same or different approaches for the predominant mechanisms underlying legal outcomes compared to those underlying health outcomes. This line of research could also consider whether there are shared or differing mediators (e.g., mental illness, risky behaviors) linking ACEs to legal system involvement versus health outcomes.

### Opportunity for Life Course Intervention

The salience of certain ACE types has important implications for child-serving providers and policymakers. With ACE screening becoming more common, there is potential to identify specific ACE exposures early and intervene to reduce risk for future legal system contact. Future work is needed to understand how to optimize the identification of high-risk youth and deliver effective secondary prevention interventions to mitigate the downstream negative sequelae of ACEs to break the link between ACE exposure and legal system involvement.

One potential opportunity to counteract the negative influence of ACEs is by investing in interventions that bolster PCE exposure across settings and life stages, such as through home visitation programs, provision of high-quality childcare or preschool, safe and supportive school environments, and supporting out-of-school time programming (California Department of Education, 2025; Centers for Disease Control and Prevention, 2019).

However, greater PCE exposure alone did not neutralize ACE-related risks, highlighting the critical need for ACE prevention and other mitigation strategies. Parental divorce/separation and childhood physical abuse were the most consistently associated with arrests and convictions, while childhood emotional and sexual abuse and parent mental health and substance use problems were inconsistently associated. Therefore, interventions that strengthen the parental unit, support parent mental and behavioral health, and prevent child abuse – especially those aimed at parental divorce/separation and physical abuse - may hold the greatest promise. Strategies that indirectly reduce the risk of parent divorce/separation include improving household financial security, access to quality childcare, family-friendly workplace policies, and healthy parenting and co-parenting support (Centers for Disease Control and Prevention, 2019). Co-parenting interventions could also be adapted and tested for use after parent divorce/separation to reduce family conflict and help parents work together (Feinberg & Kan, 2008). Other potentially useful strategies include screening for parental substance use, linking parents to community psychosocial interventions, and greater community availability of mental health and substance use treatment services (Austin et al., 2020; Chang, 2020).

These approaches should be especially responsive to the needs of girls at heightened risk of arrest. Our secondary analysis reinforces prior work that childhood sexual abuse is not only more common among females in the criminal legal system, but may amplify their risk of worse criminal legal system outcomes compared to males (Brav, 2021). Promising pathways to disrupt the “sexual abuse-to-prison pipeline” include investing in school-based sexual abuse prevention and sexual health education programs, economic supports and leadership opportunities for females, safe schools and workplaces, and youth sexual exploitation prevention practices and policies through the internet (Ali et al., 2023; Gubbels et al., 2021).

### Revisiting Conceptual Framework

The CDC’s ACE Pyramid and the Culturally-Informed ACE Framework can inform the differential findings by race and possible mediators of these relationships. Our stratified findings by race support findings of a prior study that greater cumulative ACE exposure increased the odds of reporting an arrest before age 16 for respondents who identify as Black, but not White (Fagan & Novak, 2018). Our results suggest certain ACEs may contribute to racial differences, including exposure to physical and sexual abuse, which were significantly associated with arrests exclusively among the Non-Hispanic/Latinx Black group. Additionally, this same group had stronger associations between exposure to parental divorce/separation and substance use and youth arrest.

Structural racism, such as differential criminalization of young people’s substance use and mental illness, residential segregation, and interpersonal discrimination, influences the parenting, household, and neighborhood environment in which children grow up and subsequently their degree of exposure to ACEs and PCEs (Shonkoff et al., 2021). The frameworks also describe the potential pathways through which ACEs may be linked to involvement in the criminal legal system. These include heightened risks of social, emotional, and cognitive impairments; engagement in substance use; and biopsychosocial vulnerabilities (Bernard et al., 2020; Center for Disease Control and Prevention, 2021). Additionally, racism-informed social conditions, like stress amplification, barriers to healthcare and mental health services, contribute to undermanaged mental health problems (Bernard et al., 2020; Center for Disease Control and Prevention, 2021). Studies support that substance use and undertreated mental health problems are potential mediators linking ACE exposure and legal system involvement (Folk et al., 2021).

Our PCE findings also varied by race, suggesting that existing PCE measures may be limited when applied to racially minoritized groups. We found that a nurturing relationship with both maternal and paternal figures was protective against arrests for the non-Hispanic/Latinx White but not non-Hispanic/Latinx Black youth. Greater perceived neighborhood safety was associated with higher odds of arrest for non-Hispanic/Latinx White but not non-Hispanic/Latinx Black youth. Further study is needed to understand these stratified findings by race as we noted descriptive differences in caregiver composition between non-Hispanic White and non-Hispanic Black respondents - Black respondents were about 4 times more likely to report they were not raised by a man after covariate adjustment. Besides family composition, other factors may help explain why PCEs appear to function differently for Black youth. Our PCE measure may not capture all relevant experiences; these experiences may be perceived differently across racial groups, or there may be other unmeasured factors influencing the relationship between PCE exposure and arrest history in Black youth that were not included in our study. How one defines a PCE may be influenced by contextual factors such as culture, class, generation, etc. Prior research has mostly provided descriptive insights into racial/ethnic differences in PCE reports (Crouch et al., (Crouch 2021)), but further research to assess the psychometric properties of PCE measures across racial and ethnic groups is essential to better understand PCEs in non-White populations, especially in light of the disproportionate rates of arrests and convictions among racially minoritized youth.

It is also important to keep in mind that the ACE and PCE frameworks do not fully capture the myriad of ways that structural and cultural racism, interpersonal discrimination, and other structural factors contribute to criminal legal system involvement (Lee, 2024). For example, structural racist policies and practices (e.g., historical redlining, blockbusting, urban renewal) were associated with fewer educational, socioeconomic, and health opportunities for children in one county (Blatt et al., 2024). The proposed life course interventions should occur in conjunction with efforts to dismantle racist policies and practices that contribute to differential criminal legal system contact for youth from racially marginalized backgrounds and with intersectional marginalized identities.

### Limitations

Several limitations of this study should be noted. The cross-sectional design precludes causal inferences, and overlapping timing of exposure and outcomes may allow for the possibility of reverse causality (e.g., ACEs and PCEs may have occurred after the arrests/convictions). Additionally, incarcerated individuals were excluded, but through the family-based panel design, individuals’ post-imprisonment are tracked and included. The decrease in the prevalence of arrests to convictions in our sample may have lowered our ability to detect significant associations between experiences of parental substance use and childhood sexual abuse and convictions. We also could not identify which offenses were committed, and there may be important differences in the relationship between ACE and PCE types and type of offense. Social desirability bias is a concern but was likely reduced by the self-administration of surveys. Recall bias may have influenced ACE/PCE reporting, but retrospective measurement is a standard approach in the field (Reuben et al., 2016). We adjusted for respondents’ age to reduce differential recall bias, and conducted a sensitivity analysis that restricted the age range. Although we adjusted for age and performed a subgroup analysis by age, we acknowledge that there may be other historical generational effects we were unable to fully capture (e.g., economic shifts). Given the age of the data and the respondents’ birth years, a future study is needed to test whether the observed patterns remain prominent in today’s sociopolitical and legal climate.

We note that our ACE and PCE measures were constructed from existing survey measures that were not psychometrically tested but have shown predictive validity for multiple health outcomes (La Charite et al., 2023; Schickedanz et al., 2018). These limitations to the ACE measure construction may explain why the prevalence of certain ACEs may have differed from national estimates (e.g., 22% for physical abuse vs. 10%) (Finkelhor et al., 2015). Since the survey did not ask about parental incarceration, physical neglect, presence of a non-parental adult supportive relationship, or participation in a community tradition, we were unable to include these previously recognized ACE and PCE types in the analysis. Moreover, there is no standardized methodology for calculating PCE scores; it is possible that different PCE items and methodology to construct the PCE measures could have produced different results. In this study, we did not compare the effect sizes of composite ACE and PCE scores with those of individual ACE and PCE types. Consequently, we cannot conclude whether composite multidimensional ACE and PCE risk assessments are more strongly associated with legal system outcomes than individual ACE and PCE measures. However, we anticipate that the effect sizes for individual ACE and PCE types are smaller than those observed for composite ACE and PCE scores.

We also acknowledge that reported sex, race, and ethnicity are imperfect proxies for the potential ways gender expression beyond self-reported sex and multi-level racism may influence encounters with the criminal legal system but is a limitation of the original survey. Additionally, the survey’s exclusion of Spanish-speaking respondents and post-1997 immigrants limits the generalizability of the findings to these populations. Lastly, as with any observational study, unmeasured confounding is possible.

### Future Directions

Our findings suggest that parental divorce/separation and childhood physical abuse are more strongly associated with arrests and convictions before age 26 compared to other ACE types. Future studies are needed to test whether targeted, compared to generalized ACE prevention and intervention efforts, are more effective at reducing youth arrests and convictions.

The differences in the findings by ACE types between the arrest and conviction outcomes could indicate phenotypes of young people who are arrested but not convicted of a crime. Future research could explore how ACE exposure intersects with arrests for individuals who are innocent of a crime or by the type of offense. For instance, an individual with a history of sexual abuse may be more triggered when confronted by a male police officer than a female officer, resulting in an increased likelihood of arrest? Similarly, are individuals exposed to a parent with a substance use disorder more likely to be arrested for a drug-related offense?

Moreover, future studies can explore measures of timing, duration, and severity of ACEs and PCEs to better understand their influence on young people’s criminal legal system outcomes. For instance, longitudinal studies could overcome reverse causality and measure ACEs/PCEs before arrests and convictions. Survival analysis or an equivalent methodology may be a helpful strategy to examine age at first arrest or conviction or study outcomes by juvenile versus adult legal system separately. Finally, mediation analyses may clarify pathways linking specific ACEs with early arrests and convictions among young people.

## Conclusion

In conclusion, these findings are the first to demonstrate in a nationally representative U.S. sample that certain ACEs – namely, parental divorce/separation and physical abuse – appear more closely tied to young people’s risk of arrest and conviction. In addition, we found that high exposure to PCEs overall, excluding perceived neighborhood safety, may be protective. Collectively fostering PCEs while preventing and mitigating ACEs through targeted intervention are key opportunities to disrupt young people’s pathways into the criminal legal system—a key manifestation of and driver of systemic racism—and support healthy life course trajectories.

## Abbreviations

ACE: Adverse Childhood Experience
PCE: Positive Childhood Experience
PSID: Panel Study of Income Dynamics
CRCS: Childhood Retrospective Circumstances Study
FPL: Federal Poverty Level
U.S.: United States

## References

[CR1] Aazami, A., Valek, R., Ponce, A. N., Zare, H., Aazami, A., Valek, R., Ponce, A. N., & Zare, H. (2023). Risk and protective factors and interventions for reducing juvenile delinquency: A systematic review. *Social Sciences*. 10.3390/socsci12090474

[CR2] Abrams, L. S., Mizel, M. L., & Barnert, E. S. (2021). The criminalization of young children and overrepresentation of Black youth in the juvenile justice system. *Race and Social Problems,**13*(1), 73–84. 10.1007/s12552-021-09314-7

[CR3] Ali, S., Haykal, H. A., & Youssef, E. Y. M. (2023). Child sexual abuse and the internet—A systematic review. *Human Arenas,**6*(2), 404–421. 10.1007/s42087-021-00228-9

[CR4] Austin, A. E., Lesak, A. M., & Shanahan, M. E. (2020). Risk and protective factors for child maltreatment: A review. *Current Epidemiology Reports,**7*, 334–342. 10.1007/s40471-020-00252-3/Published 34141519 10.1007/s40471-020-00252-3 PMC8205446

[CR5] Baglivio, M. T., & Wolff, K. T. (2021). Positive childhood experiences (PCE): Cumulative resiliency in the face of adverse childhood experiences. *Youth Violence and Juvenile Justice,**19*(2), 139–162. 10.1177/1541204020972487

[CR6] Baglivio, M. T., Epps, N., Swartz, K., Huq, M. S., Sheer, A., & Hardt, N. (2014a). The prevalence of adverse childhood experiences (ACE) in the lives of juvenile offenders. *Journal of Juvenile Justice*, *3*(2).

[CR7] Baglivio, M. T., Jackowski, K., Greenwald, M. A., & Howell, J. C. (2014). Serious, violent, and chronic juvenile offenders. *Criminology and Public Policy,**13*(1), 83–116. 10.1111/1745-9133.12064

[CR8] Barnert, E. S., Perry, R., Shetgiri, R., Steers, N., Dudovitz, R., Heard-Garris, N. J., Zima, B., & Chung, P. J. (2021). Adolescent protective and risk factors for incarceration through early adulthood. *Journal of Child and Family Studies,**30*(6), 1428–1440. 10.1007/s10826-021-01954-y

[CR9] Barnert, E. S., Schlichte, L. M., Tolliver, D. G., La Charite, J., Biely, C., Dudovitz, R., Leifheit, K., Russ, S., Sastry, N., Yama, C., Slavich, G. M., & Schickedanz, A. (2023). Parents’ adverse and positive childhood experiences and offspring involvement with the criminal legal system. *JAMA Network Open,**6*(10), Article E2339648. 10.1001/jamanetworkopen.2023.39648 37878312 10.1001/jamanetworkopen.2023.39648 PMC10600584

[CR10] Basto-Pereira, M., Miranda, A., Ribeiro, S., & Maia, Â. (2016). Growing up with adversity: From juvenile justice involvement to criminal persistence and psychosocial problems in young adulthood. *Child Abuse & Neglect*, *62*, 63–75. 10.1016/j.chiabu.2016.10.011 27794243 10.1016/j.chiabu.2016.10.011

[CR11] Beaule, A., Brown, C., Campbell, F., Dascola, M., Freedman, V., Insolera, N., Pfeffer, F., Mcgonagle, K., Sastry, N., Schlegel, J., Simmert, B., & Warra, J. (2015). *PSID main interview user manual: Release 2015* (pp. 1–63). University of Michigan. https://psidonline.isr.umich.edu/data/Documentation/UserGuide2013.pdf

[CR12] Behrman, J. R., & Taubman, P. (1990). The intergenerational correlation between children’s adult earnings and their parents’ income: Results from the Michigan Panel Survey of Income Dynamics. *Review of Income and Wealth,**36*(2), 115–127. 10.1111/j.1475-4991.1990.tb00275.x

[CR13] Bernard, D. L., Calhoun, C. D., Banks, D. E., Halliday, C. A., Hughes-Halbert, C., & Danielson, C. (2020). Making the “C-ACE” for a culturally-informed adverse childhood experiences framework to understand the pervasive mental health impact of racism on Black youth. *Journal of Child & Adolescent Trauma,**14*, 233–247. 10.1007/s40653-020-00319-9 33986909 10.1007/s40653-020-00319-9 PMC8099967

[CR14] Bethell, C., Jones, J., Gombojav, N., Linkenbach, J., & Sege, R. (2019). Positive childhood experiences and adult mental and relational health in a statewide sample: Associations across adverse childhood experiences levels. *JAMA Pediatrics*. 10.1001/jamapediatrics.2019.3007 31498386 10.1001/jamapediatrics.2019.3007 PMC6735495

[CR15] Blatt, L. R., Sadler, R. C., Jones, E. J., Miller, P., Hunter-Rue, D. S., & Votruba-Drzal, E. (2024). Historical structural racism in the built environment and contemporary children’s opportunities. *Pediatrics,**153*(2), Article e2023063230. 10.1542/peds.2023-063230 38192230 10.1542/peds.2023-063230

[CR16] Brav, A. (2021). Abuse-to-prison pipeline: A call for a human rights approach. *Journal of Aggression, Maltreatment & Trauma,**30*(3), 279–293. 10.1080/10926771.2021.1874584

[CR17] Brock, R. L., & Kochanska, G. (2016). Interparental conflict, children’s security with parents, and long-term risk of internalizing problems: A longitudinal study from ages 2 to 10. *Development and Psychopathology,**28*(1), 45–54. 10.1017/S0954579415000279 25797703 10.1017/S0954579415000279 PMC4580501

[CR18] Broidy, L., & Agnew, R. (1997). Gender and crime: A general strain theory perspective. *Journal of Research in Crime and Delinquency*, *34*(3), 275–306. 10.1177/0022427897034003001

[CR19] Brummer, J., Hesse, M., Frederiksen, K. S., Karriker-Jaffe, K. J., & Bloomfield, K. (2021). How do register-based studies contribute to our understanding of alcohol’s harms to family members? A scoping review of relevant literature. *Journal of Studies on Alcohol and Drugs*, *82*(4), 445–456. 10.15288/jsad.2021.82.445 34343075

[CR20] California Department of Education (2025, February 16). *Safe and supportive schools—School environment*. https://www.cde.ca.gov/ls/ss/se/safesupportive.asp

[CR21] Center for Disease Control and Prevention (2021, April 6). *Violence prevention: About the CDC-Kaiser ACE study*. https://www.cdc.gov/violenceprevention/aces/about.html

[CR22] Centers for Disease Control and Prevention (2019). *Adverse childhood experiences prevention resources for action*. *Violence Prevention*. https://www.cdc.gov/violenceprevention/pdf/aces-prevention-resource_508.pdf

[CR23] Chang, G. (2020). Maternal substance use: Consequences, identification, and interventions. *Alcohol Research: Current Reviews*. 10.35946/arcr.v40.2.06 32612898 10.35946/arcr.v40.2.06 PMC7304408

[CR24] Chang, X., Jiang, X., Mkandarwire, T., & Shen, M. (2019). Associations between adverse childhood experiences and health outcomes in adults aged 18–59 years. *PLoS One,**14*(2), Article e0211850. 10.1371/JOURNAL.PONE.0211850 30730980 10.1371/journal.pone.0211850 PMC6366931

[CR25] Chesney-Lind, M., & Merlo, A. V. (2015). Global war on girls? Policing girls’ sexuality and criminalizing their victimization. *Women & Criminal Justice,**25*(1–2), 71–82. 10.1080/08974454.2015.1026776

[CR26] Craig, J. M., Piquero, A. R., Farrington, D. P., & Ttofi, M. M. (2017). A little early risk goes a long bad way: Adverse childhood experiences and life-course offending in the Cambridge study. *Journal of Criminal Justice,**53*, 34–45. 10.1016/j.jcrimjus.2017.09.005

[CR27] Essex, M. J., Klein, M. H., Cho, E., & Kraemer, H. C. (2003). Exposure to maternal depression and marital conflict: Gender differences in children’s later mental health symptoms. *Journal of the American Academy of Child & Adolescent Psychiatry,**42*(6), 728–737. 10.1097/01.CHI.0000046849.56865.1D 12921481 10.1097/01.CHI.0000046849.56865.1D

[CR28] Fagan, A. A., & Novak, A. (2018). Adverse childhood experiences and adolescent delinquency in a high-risk sample: A comparison of White and Black youth. *Youth Violence and Juvenile Justice,**16*(4), 395–417. 10.1177/1541204017735568

[CR29] Farrington, D. P., Ttofi, M. M., & Piquero, A. R. (2016). Risk, promotive, and protective factors in youth offending: Results from the Cambridge study in delinquent development. *Journal of Criminal Justice,**45*, 63–70. 10.1016/j.jcrimjus.2016.02.014

[CR30] Feinberg, M. E., & Kan, M. L. (2008). Establishing family foundations: Intervention effects on coparenting, parent/infant well-being, and parent-child relations. *Journal of Family Psychology,**22*(2), 253–263. 10.1037/0893-3200.22.2.253 18410212 10.1037/0893-3200.22.2.253 PMC3178882

[CR31] Felitti, V. J. (2002). The relation between adverse childhood experiences and adult health: Turning gold into lead. *The Permanente Journal,**6*(1), 44–47. 30313011 10.7812/tpp/02.994 PMC6220625

[CR32] Felitti, V. J., Anda, R. F., Nordenberg, D., Williamson, D. F., Spitz, A. M., Edwards, V., Koss, M. P., & Marks, J. S. (1998). Relationship of childhood abuse and household dysfunction to many of the leading causes of death in adults: The adverse childhood experiences (ACE) study. *American Journal of Preventive Medicine,**14*(4), 245–258. 10.1016/S0749-3797(98)00017-8 9635069 10.1016/s0749-3797(98)00017-8

[CR33] Finkelhor, D., Shattuck, A., Turner, H., & Hamby, S. (2015). A revised inventory of adverse childhood experiences. *Child Abuse & Neglect,**48*, 13–21. 10.1016/J.CHIABU.2015.07.011 26259971 10.1016/j.chiabu.2015.07.011

[CR34] Folk, J. B., Ramos, L. M. C., Bath, E. P., Rosen, B., Marshall, B. D. L., Kemp, K., Brown, L., Conrad, S., & Tolou-Shams, M. (2021). The prospective impact of adverse childhood experiences on justice-involved youth’s psychiatric symptoms and substance use. *Journal of Consulting and Clinical Psychology,**89*(6), 483–498. 10.1037/ccp0000655 34264697 10.1037/ccp0000655 PMC8754104

[CR35] Graf, G.-J., Chihuri, S., Blow, M., & Li, G. (2021). Adverse childhood experiences and criminal justice contact in adulthood. *Pediatrics,**147*(1), Article e2020021030. 10.1542/peds.2020-021030 33328338 10.1542/peds.2020-021030 PMC7786827

[CR36] Gubbels, J., van der Put, C. E., Stams, G. J. J. M., & Assink, M. (2021). Effective components of school-based prevention programs for child abuse: A meta-analytic review. *Clinical Child and Family Psychology Review,**24*(3), 553–578. 10.1007/s10567-021-00353-5 34086183 10.1007/s10567-021-00353-5 PMC8176877

[CR37] Guo, S., O’Connor, M., Mensah, F., Olsson, C. A., Goldfeld, S., Lacey, R. E., Slopen, N., Thurber, K. A., & Priest, N. (2021). Measuring positive childhood experiences: Testing the structural and predictive validity of the health outcomes from positive experiences (HOPE) framework. *Academic Pediatrics*, *22*(6), 942–951. 10.1016/j.acap.2021.11.003 34801761 10.1016/j.acap.2021.11.003

[CR38] Huang, C. X., Halfon, N., Sastry, N., Chung, P. J., & Schickedanz, A. (2023). Positive childhood experiences and adult health outcomes. *Pediatrics,**152*(1), Article e2022060951. 10.1542/peds.2022-060951 37337829 10.1542/peds.2022-060951 PMC10312234

[CR39] Hughes, K., Bellis, M. A., Hardcastle, K. A., Sethi, D., Butchart, A., Mikton, C., Jones, L., & Dunne, M. P. (2017). The effect of multiple adverse childhood experiences on health: A systematic review and meta-analysis. *The Lancet Public Health*, *2*(8), e356–e366. 10.1016/S2468-2667(17)30118-4 29253477 10.1016/S2468-2667(17)30118-4

[CR40] Johnson, R. J., Ross, M. W., Taylor, W. C., Williams, M. L., Carvajal, R. I., & Peters, R. J. (2006). Prevalence of childhood sexual abuse among incarcerated males in county jail. *Child Abuse & Neglect,**30*(1), 75–86. 10.1016/j.chiabu.2005.08.013 16412506 10.1016/j.chiabu.2005.08.013

[CR41] Jones, M. S., & Pierce, H. (2021). Early exposure to adverse childhood experiences and youth delinquent behavior in fragile families. *Youth & Society,**53*(5), 841–867. 10.1177/0044118X20908759

[CR42] Jordan, K. L., & Gabbidon, S. L. (2010). Race/ethnicity and perceptions of safety among a national sample of Americans. *Criminal Justice Review,**35*(3), 281–294. 10.1177/0734016810366453

[CR43] King, S. M., Keyes, M., Malone, S. M., Elkins, I., Legrand, L. N., Iacono, W. G., & McGue, M. (2009). Parental alcohol dependence and the transmission of adolescent behavioral disinhibition: A study of adoptive and non-adoptive families. *Addiction,**104*(4), 578–586. 10.1111/j.1360-0443.2008.02469.x 19215604 10.1111/j.1360-0443.2008.02469.x PMC2751628

[CR44] Kruzan, S., & Lesley, A. (2025). *Unheard: The epidemic of severe childhood trauma among girls rried as adults* (pp. 1–32). The Gault Center. https://humanrightsforkids.org/publication/unheard-the-epidemic-of-severe-childhood-trauma-among-girls-tried-as-adults/

[CR45] La Charite, J., Khan, M., Dudovitz, R., Nuckols, T., Sastry, N., Huang, C., Lei, Y., & Schickedanz, A. (2023). Specific domains of positive childhood experiences (PCEs) associated with improved adult health: A nationally representative study. *SSM - Population Health,**24*, Article 101558. 10.1016/j.ssmph.2023.101558 38034480 10.1016/j.ssmph.2023.101558 PMC10685007

[CR46] Lee, H. (2024). How does structural racism operate (in) the contemporary US criminal justice system? *Annual Review of Criminology,**7*, 233–255. 10.1146/annurev-criminol-022422-015019

[CR47] Lesley, A., & Pierre, S. S. L. (2025). *The childhood trauma-to-prison pipeline* (pp. 1–120). Human Rights for Kids. https://humanrightsforkids.org/wp-content/uploads/The-Childhood-Trauma-to-Prison-Pipeline.pdf

[CR48] Lockwood, A., Peck, J. H., Wolff, K. T., & Baglivio, M. T. (2022). Understanding adverse childhood experiences and juvenile court outcomes: The moderating role of race and ethnicity. *Youth Violence and Juvenile Justice,**20*, 83–112. 10.1177/15412040211063437

[CR49] McGill, K. A., & Stefurak, T. (2021). “Man up”: Sex-Differentiated Pathways of Juvenile Delinquency through Trauma, Borderline Traits & Offense Patterns. *Juvenile and Family Court Journal,**72*(3), 37–65. 10.1111/jfcj.12207

[CR50] McGonagle, K., & Freedman, V. (2015, September). *The Panel Study of Income Dynamics’ Childhood Retrospective Circumstance Study (PSID-CRCS) user guide: Final release 1*. Institute for Social Research, University of Michigan. https://psidonline.isr.umich.edu/CRCS/2014UserGuide.pdf

[CR51] Mersky, J. P., Choi, C., Plummer Lee, C., & Janczewski, C. E. (2021). Disparities in adverse childhood experiences by race/ethnicity, gender, and economic status: Intersectional analysis of a nationally representative sample. *Child Abuse & Neglect,**117*, Article 105066. 10.1016/j.chiabu.2021.105066 33845239 10.1016/j.chiabu.2021.105066

[CR52] Mueller, K. C., & Carey, M. T. (2022). How positive and negative childhood experiences interact with resiliency theory and the general theory of crime in juvenile probationers. *Youth Violence and Juvenile Justice*. 10.1177/15412040221131278

[CR53] Narayan, A. J., Rivera, L. M., Bernstein, R. E., Harris, W. W., & Lieberman, A. F. (2018). Positive childhood experiences predict less psychopathology and stress in pregnant women with childhood adversity: A pilot study of the benevolent childhood experiences (BCEs) scale. *Child Abuse & Neglect,**78*, 19–30. 10.1016/J.CHIABU.2017.09.022 28992958 10.1016/j.chiabu.2017.09.022

[CR54] Nellis, A. (2024). *Mass incarceration trends*. The Sentencing Project. https://www.sentencingproject.org/reports/mass-incarceration-trends/

[CR55] *New in Stata 18 | Stata*. (2024). https://www.stata.com/new-in-stata/

[CR56] Niolon, P. H., Kearns, M., Dillis, J., Rambo, Kristen, Irving, S., Armstead, T., & Gilbert, L. (2017). *Preventing intimate partner violence across the lifespan: A technical package of programs, policies, and practices*. National Center for Injury Prevention and Control (U.S. https://stacks.cdc.gov/view/cdc/45820 Division of Violence Prevention.

[CR57] Novak, A. S. (2025). Pathways of punishment: Racial differences in the relationships between ACEs and behavioral and punitive consequences. *American Journal of Criminal Justice*. 10.1007/s12103-025-09820-z

[CR58] Nuytiens, A., & Christiaens, J. (2016). Female pathways to crime and prison: Challenging the (US) gendered pathways perspective. *European Journal of Criminology*, *13*(2), 195–213. 10.1177/1477370815608879

[CR59] Petruccelli, K., Davis, J., & Berman, T. (2019). Adverse childhood experiences and associated health outcomes: A systematic review and meta-analysis. *Child Abuse & Neglect,**97*, Article 104127. 10.1016/J.CHIABU.2019.104127 31454589 10.1016/j.chiabu.2019.104127

[CR60] Poole, M. K., Seal, D. W., & Taylor, C. A. (2014). A systematic review of universal campaigns targeting child physical abuse prevention. *Health Education Research,**29*(3), 388–432. 10.1093/her/cyu012 24711483 10.1093/her/cyu012 PMC4021196

[CR61] Reuben, A., Moffitt, T. E., Caspi, A., Belsky, D. W., Harrington, H., Schroeder, F., Hogan, S., Ramrakha, S., Poulton, R., & Danese, A. (2016). Lest we forget: Comparing retrospective and prospective assessments of adverse childhood experiences in the prediction of adult health. *Journal of Child Psychology and Psychiatry,**57*(10), 1103–1112. 10.1111/jcpp.12621 27647050 10.1111/jcpp.12621 PMC5234278

[CR62] Schickedanz, A., Halfon, N., Sastry, N., & Chung, P. J. (2018). Parents’ adverse childhood experiences and their children’s behavioral health problems. *Pediatrics*, *142*(2). 10.1542/PEDS.2018-0023/76794 10.1542/peds.2018-0023 PMC6317990 29987168

[CR63] Shaefer, H. L., Lapidos, A., Wilson, R., & Danziger, S. (2018). Association of income and adversity in childhood with adult health and well-being. *Social Service Review*. 10.1086/696891

[CR64] Shonkoff, J. P., Slope, N., & Williams, D. R. (2021). Early childhood adversity, toxic stress, and the impacts of racism on the foundations of health. *Annual Review of Public Health,**42*, 115–134. 10.1146/annurev-publhealth-090419-101940 33497247 10.1146/annurev-publhealth-090419-101940

[CR65] Sivertsson, F., Carlsson, C., & Hoherz, A. (2023). Is there a long-term criminogenic effect of the exposure to a paternal conviction during upbringing? An analysis of full siblings using Swedish register data. *Journal of Quantitative Criminology*, *39*(1), 53–73. 10.1007/s10940-021-09529-2

[CR66] Slopen, N., Shonkoff, J. P., Albert, M. A., Yoshikawa, H., Jacobs, A., Stoltz, R., & Williams, D. R. (2016). Racial disparities in child adversity in the U.S.: Interactions with family immigration history and income. *American Journal of Preventive Medicine*, *50*(1), 47–56. 10.1016/j.amepre.2015.06.013 26342634 10.1016/j.amepre.2015.06.013

[CR67] Sparks, L. A., Trentacosta, C. J., Hicks, M. R., Kernsmith, P., & Smith-Darden, J. (2021). Hope as a protective factor: Relations to adverse childhood experiences, delinquency, and posttraumatic stress symptoms. *Journal of Child and Family Studies,**30*(12), 3005–3015. 10.1007/s10826-021-02119-7

[CR68] Spivak, A. L., Wagner, B. M., Whitmer, J. M., & Charish, C. L. (2014). Gender and status offending: Judicial paternalism in juvenile justice processing. *Feminist Criminology,**9*(3), 224–248. 10.1177/1557085114531318

[CR69] STROBE (2023). *STROBE Strengthening the reporting of observational studies in epidemiology*. https://www.strobe-statement.org/

[CR70] Sweeten, G., Piquero, A. R., & Steinberg, L. (2013). Age and the explanation of crime, revisited. *Journal of Youth and Adolescence*, *42*(6), 921–938. 10.1007/s10964-013-9926-4 23412690 10.1007/s10964-013-9926-4

[CR71] Testa, A., Jackson, D. B., Ganson, K. T., & Nagata, J. M. (2022). Adverse childhood experiences and criminal justice contact in adulthood. *Academic Pediatrics,**22*(6), 972–980. 10.1016/j.acap.2021.10.011 34752957 10.1016/j.acap.2021.10.011

[CR72] Thompson, H. A. (2010). Why mass incarceration matters: Rethinking crisis, decline, and transformation in postwar American history. *Journal of American History,**97*(3), 703–734.

[CR73] Tolliver, D. G., Abrams, L. S., Biely, C., Meza, B. P. L., Schickedanz, A., Guerrero, A. D., Jackson, N. J., Bath, E., Heard-Garris, N., Dudovitz, R., & Barnert, E. (2023). United States youth arrest and health across the life course: A nationally representative longitudinal study. *Academic Pediatrics,**23*(4), 722–730. 10.1016/j.acap.2022.08.009 36055448 10.1016/j.acap.2022.08.009 PMC9971348

[CR74] White, H. R., & Frisch-Scott, N. E. (2023). Childhood victimization and adult incarceration: A review of the literature. *Trauma, Violence, & Abuse,**24*(3), 1543–1559. 10.1177/15248380211073841 10.1177/15248380211073841 35354348

[CR75] Wilens, T. E., & Rosenbaum, J. F. (2013). Transitional aged youth: A new frontier in child and adolescent psychiatry. *Journal of the American Academy of Child and Adolescent Psychiatry,**52*(9), 887–890. 10.1016/j.jaac.2013.04.020 23972688 10.1016/j.jaac.2013.04.020

[CR76] Yohros, A. (2023). Examining the relationship between adverse childhood experiences and juvenile recidivism: A systematic review and meta-analysis. *Trauma, Violence & Abuse,**24*(3), 1640–1655. 10.1177/15248380211073846 10.1177/15248380211073846 35166600

[CR77] Zahn, M. A., Agnew, R., Fishbein, D., Miller, S., Dakoff, G., Kruttschnitt, C., Giordano, P., Gottfredson, D. C., Payne, A. A., Feld, B. C., & Chesney-Lind, M. (2010). Girls study group understanding and responding to girls’ delinquency: Causes and correlates of girls’ delinquency. *Office of Justice Programs U S Department of Justice*. https://www.ojp.gov/pdffiles1/ojjdp/226358.pdf

[CR78] Crouch, E., Radcliff, E., Merrell, M. A., Brown, M. J., Ingram, L. A., & Probst, J. (2021). Racial/ethnic differences in positive childhood experiences across a national sample. Child abuse & neglect, 115, 105012. 10.1016/j.chiabu.2021.105012 33639558

